# Robust cross-cohort gut microbiome associations with COVID-19 severity

**DOI:** 10.1080/19490976.2023.2242615

**Published:** 2023-08-07

**Authors:** Junhui Li, Tarini Shankar Ghosh, Rachel McCann, Patrick Mallon, Colin Hill, Lorraine Draper, David Schult, Liam J. Fanning, Robert Shannon, Corinna Sadlier, Mary Horgan, Liam O’Mahony, Paul W. O’Toole

**Affiliations:** a School of Microbiology, University College Cork, Cork, Ireland; b APC Microbiome Ireland, University College Cork, Cork, Ireland; c Centre for Experimental Pathogen Host Research, School of Medicine, University College Dublin, St Vincent’s University Hospital, Dublin, Ireland; d Department of Internal Medicine II, Klinikum Rechts der Isar, Technical University of Munich, School of Medicine, Munich, Germany; e Department of Medicine, University College Cork, Cork, Ireland; f Department of Infectious Diseases, Cork University Hospital, Cork, Ireland

**Keywords:** COVID, gut microbiome, meta-analysis, disease severity, microbiota-targeted diet, eukaryotic microorganisms

## Abstract

Although many recent studies have examined associations between the gut microbiome and COVID-19 disease severity in individual patient cohorts, questions remain on the robustness across international cohorts of the biomarkers they reported. Here, we performed a meta-analysis of eight shotgun metagenomic studies of COVID-19 patients (comprising 1,023 stool samples) and 23 > 16S rRNA gene amplicon sequencing (16S) cohorts (2,415 total stool samples). We found that disease severity (as defined by the WHO clinical progression scale) was associated with taxonomic and functional microbiome differences. This alteration in gut microbiome configuration peaks at days 7–30 post diagnosis, after which the gut microbiome returns to a configuration that becomes more similar to that of healthy controls over time. Furthermore, we identified a core set of species that were consistently associated with disease severity across shotgun metagenomic and 16S cohorts, and whose abundance can accurately predict disease severity category of SARS-CoV-2 infected subjects, with *Actinomyces oris* abundance predicting population-level mortality rate of COVID-19. Additionally, we used relational diet-microbiome databases constructed from cohort studies to predict microbiota-targeted diet patterns that would modulate gut microbiota composition toward that of healthy controls. Finally, we demonstrated the association of disease severity with the composition of intestinal archaeal, fungal, viral, and parasitic communities. Collectively, this study has identified robust COVID-19 microbiome biomarkers, established accurate predictive models as a basis for clinical prognostic tests for disease severity, and proposed biomarker-targeted diets for managing COVID-19 infection.

## Introduction

Coronavirus disease 2019 (COVID-19), an extremely contagious viral disease, has resulted in near 6.9 million deaths globally as of 7 March 2023.^1^ SARS-CoV-2-induced inflammation is an important component of COVID-19 disease severity.^2–4^ Elevated levels of inflammatory markers such as C-reactive protein and inflammatory cytokines are linked to the severity of SARS-CoV-2 infection, with several immune markers remaining elevated for long periods following infection.^2,5–9^ Gastrointestinal (GI) symptoms are common (~50%) in patients with COVID‐19^10,11^ and 6 months later, with the prevalence of 29.4% and 43.8% in two recent surveys.^12,13^ GI symptoms are associated with more severe disease,^14–16^ though not with higher risk of mortality.^16,17^ The gut microbiome, recognized as being pivotal for immune education, protection from infection and preventing excessive inflammatory responses,^18,19^ is significantly altered in patients with COVID-19, and gut microbiome alteration or dysbiosis can persist in patients with COVID-19 for manymonths.^20–25^ There are significant correlations between changes in the gut microbiome and host immune and metabolic responses that are important for successful outcomes to acute SARS-CoV-2 infection, and the long-term sequelae termed post-COVID condition or long COVID.^15,21,26–28^

It is reasonable to propose that gut microbiome restoration might mitigate symptoms in patients with acute or long COVID.^15,29^ Long COVID includes a spectrum of symptoms such as fatigue, shortness of breath, and impaired cognitive function.^30^ There are reports suggesting that alterations in gut and airway microbiomes may play a role in long COVID neurological symptoms.^31,32^ Establishing a clear framework of taxa that are robustly differentially abundant in COVID infection across multiple cohorts is necessary to progress this theory. Recent studies have also suggested a strong association between COVID-19 disease severity and the gut microbiome,^20–24,26^ but it remains unclear whether there are highly reproducible disease severity-associated microbial biomarkers across different cohorts in distinct geographies. Thus, larger and diverse cohorts are urgently needed to provide robust and generalizable microbial signatures associated with COVID-19 disease.

**Figure 1.**
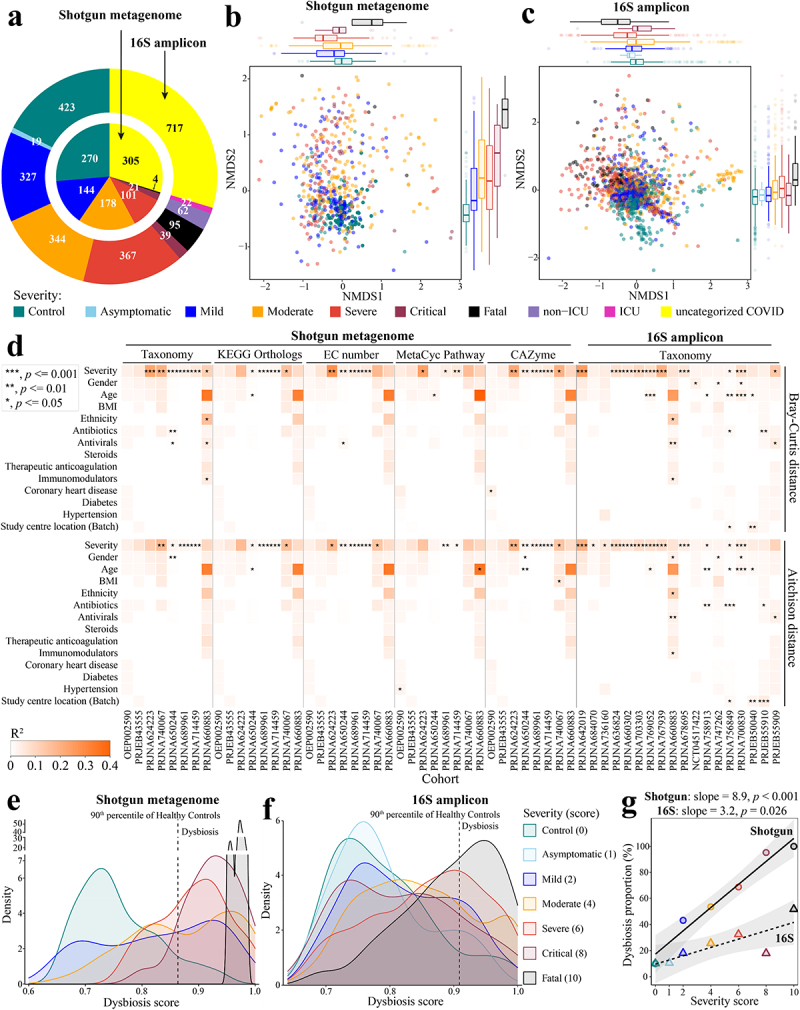
Altered gut microbiome of COVID-19 patients is associated with disease severity. a. Distribution of disease severity categories across eight shotgun metagenome cohorts (inner circle) and twenty-three 16S rRNA gene amplicon sequencing cohorts (outer circle). Non-metric multidimensional scaling (NMDS) of microbiome-severity correlation based on species-level bray-curtis dissimilarity of B pooled shotgun metagenomes (*n* = 718) and C pooled 16S rRNA gene amplicon sequencing samples (*n* = 1,698) with known disease severity (See Extended Data Fig. S1a,b for the same analysis but including uncategorized COVID-19 samples, and Extended Data Fig. S2 and Fig. S3 for individual cohorts, respectively); colored boxplots on the top and the right represent Bray-Curtis dissimilarity by disease severity in the first and second ordinations respectively. d. COVID disease severity is significantly associated with differences in the microbiome i.e. taxonomy and function (KO, EC, pathway, and CAZyme) across cohorts (first stool sample only from each subject); taxonomy analysis is based on Bray-Curtis dissimilarity matrices computed on relative abundance, and Aitchison dissimilarity matrices computed on centered log-ratio (CLR) transformed absolute abundance with covariate adjusted and marginal sums of squares applied where appropriate. Global distribution of microbial dysbiosis scores as a measure of disease activity based on e shotgun metagenomes with known disease severity and f. 16S rRNA gene amplicon sequencing samples with known disease severity (See Extended Data Fig. S4a,b for the this analysis including uncategorized COVID-19 samples). g. Microbiome dysbiosis proportion is positively related to disease severity (linear model; data underlying this plot can be found at Extended Data Fig. S4a,b).

To study COVID-associated changes in the gut microbiome, we performed a meta-analysis of eight shotgun metagenomic studies (totaling 1,023 gut samples from 270 healthy controls and 753 COVID-19 cases) and 23 16S rRNA gene amplicon sequencing (16S) cohorts (totaling 2,415 gut samples from 423 healthy controls and 1,992 cases) of COVID-19 (Figure 1a, see Methods for inclusion criteria). We identified microbiome covariates with disease severity, microbiome dynamics within-subject, diagnostic biomarkers for outcomes in patients with COVID-19, dietary ingredients that might ameliorate clinical outcomes by modulating the microbiome, and gut microbiome alterations within the archaea, fungi, viruses, and parasites.

## Results

### COVID-19 gut microbiome dataset assembly

In this meta-analysis, we collected microbiome data from eight shotgun metagenomic cohorts and 23 16S cohorts of COVID-19 gut microbiome (Table S1) that had been published by April 2022. These studies encompassed a large range of disease severity (asymptomatic, mild, moderate, severe, critical, fatal, and healthy control; Figure 1a), anthropometrics (e.g. age, sex, body mass index (BMI) and ethnicity), geography (12 countries across four continents), phase of infection (acute and convalescent), frequency and length of longitudinal sampling (~9 months), medication (e.g. antibiotics, antivirals, steroids, immunomodulators, and anticoagulants), and comorbidities (e.g. coronary heart disease, diabetes, and hypertension). Importantly, in all cohorts, we determined microbiome differences while adjusting for other covariates. To ensure consistency in the bioinformatic analyses, all metagenomic sequencing data were reprocessed using MetaPhlAn 3 for taxonomic profiling and HUMAnN 3 for functional profiling,^33^ while all 16S data were reprocessed using SPINGO for taxonomic profiling^34^ (please see Methods for details).

### Altered gut microbiome in patients with COVID-19 is associated with disease severity

As described in Methods, disease severity was defined according to WHO criteria, from healthy control through to critically ill, or fatally ill. Analysis of the Bray-Curtis ordination of species-level taxonomic composition determined for pooled shotgun metagenomic datasets revealed that disease severity significantly associated with the first and second axes of variation, respectively (Envfit, r^2^ = 0.094 and 0.218, *p* < .001 and .001, *n* = 718, Figure 1b). An association with disease severity was also observed for the Bray-Curtis ordination of 16S amplicon sequence data (Envfit, r^2^ = 0.300 and 0.165, *p* < .001 and .001, *n* = 1698, Figure 1c). We further investigated if the difference exists between different severities in each ordination. For shotgun metagenomic data (Figure 1b), 33.3% (5/15) and 73.3% (11/15) of comparisons were significantly different on NMDS1 and NMDS2, respectively, and for 16S data (Figure 1c), 76.2% (16/21) on NMDS1 and 66.7% (14/21) on NMDS2 (Wilcoxon rank-sum test,*p* < .05, Table S2a). However, datasets exhibited strong cohort effects in the two-dimensional ordination (Envfit; shotgun: r^2^ = 0.212, *p* < .001; 16S:r^2^ = 0.397, *p* < .001). This apparent microbiome separation can be misleading because covariates in studies may confound associations between disease severity and microbiome composition. We therefore assessed microbiome composition variation that associated with COVID-19 disease severity while accounting for confounding factors across cohorts (Figure 1d, Extended Data Fig. S2 and Extended Data Fig. S3). Although numerous other factors such as age, gender, medication, other comorbidities, and study center location were significantly associated with the microbiome in some cases, COVID-19 disease severity was the strongest association with the microbiome among the factors tested/available in both the shotgun- and 16S-based gut taxonomic compositions across cohorts, irrespective of testing either Bray-Curtis dissimilarity based on relative abundance (PERMANOVA,*p* < .05 in six out of eight shotgun cohorts and 10 out of 18 16S cohorts, respectively) or Aitchison dissimilarity based on centered-log-ratio-transformed (CLR) absolute abundance (PERMANOVA, *p* < .05 in four out of eight shotgun cohorts and 12 out of 18 16S cohorts, respectively) (Figure 1d, Table S2b). In addition, we assessed four aspects of microbiome function predicted for the shotgun metagenomic datasets: KEGG Orthologs (KOs),^35^ Enzyme Commission number (EC number), MetaCyc pathway (Pathway),^36^ and carbohydrate active enzymes (CAZymes).^37^ This analysis identified significant levels of disease severity-associated microbiome variation in, on average, half of the cohorts when adjusting for confounding factors (PERMANOVA, *p* < .05, r^2^ = 0.020–0.277 (Bray-Curtis dissimilarity) and 0.015–0.183 (Aitchison dissimilarity), Figure 1d, Table S2b). Thus, the composition and inferred function of the gut microbiome derived from shotgun metagenomic or 16S data both correlated with disease severity.

Next, we quantified the degree of microbiome dysbiosis by classifying as ‘dysbiotic’ datasets with taxonomic compositions beyond the 90^th^ percentile of the distribution in healthy controls, in accordance with the classification procedure proposed by.^38^ Notably, the frequency of pooled shotgun metagenomes classified as dysbiotic was strongly associated with COVID-19 disease severity (Spearman: ρ = 1, *p* = 0.0028; Figure 1e,g, Extended Data Fig. S4a). Although the dysbiosis proportion was lower in the pooled 16S amplicon datasets compared to that of the shotgun metagenomes (i.e. flatter slope for the linear model of the 16S data, Figure 1g; Extended Data Fig. S4a,b), the dysbiosis proportion of the pooled 16S amplicon samples was also significantly related to COVID-19 disease severity (Spearman: ρ = 0.79, *p* = 0.048; Figure 1g, Extended Data Fig. S4b).

Given the limitations of quantifying the global microbiome dysbiosis using this approach, we further investigated the dysbiosis frequency within each cohort. Like the pooled analysis, the dysbiosis proportion of shotgun metagenomes within individual cohorts generally increased as a function of COVID-19 disease severity, and with 100% of metagenomes from COVID-19 patients who died or suffered from critical, severe, or even mild disease classified as dysbiotic in four cohorts (Extended Data Fig. S4c). COVID-19 disease severity did not always correlate strictly with the dysbiosis proportion of 16S amplicon samples within individual cohorts, e.g., PRJNA758913^39^ and PRJEB50040,^22^ probably due to the small sample size of the 16S-based studies with disease severity data, or comparison to global healthy controls (Extended Data Fig. S4c). However, the generally significant association provides further evidence that gut microbiome alterations are particularly relevant in COVID-19. Together, these patterns suggest that disease severity-associated differences in the gut microbiome are robustly detectable across very disparate geographical locations. We discuss particular species differences below.

### The altered gut microbiome of COVID-19 patients becomes more similar to that of healthy controls over time

**Figure 2.**
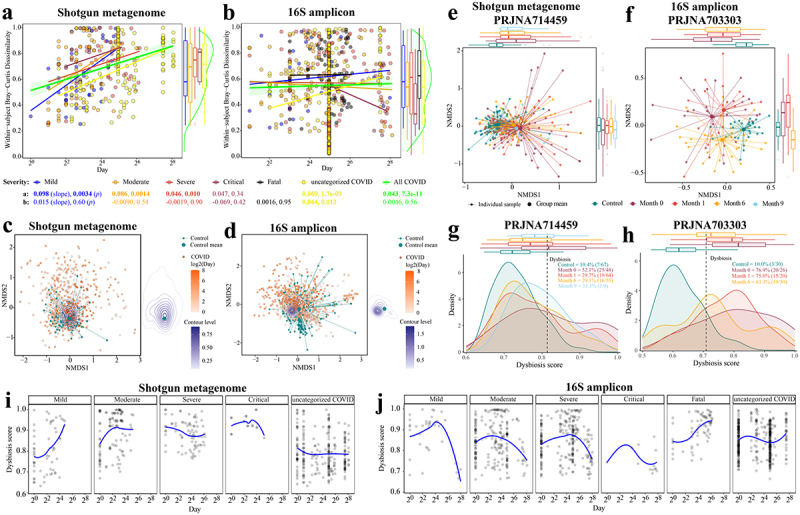
Longitudinal analysis reveals that the gut microbiome in patients with COVID-19 shifts back toward a state more similar to healthy controls over time. Within-subject Bray-Curtis dissimilarity of gut microbiome in COVID-19 individuals from (a) pooled shotgun metagenomes (*n* = 310 samples from 165 patients) and (b) pooled 16S amplicon samples (*n* = 594 samples from 387 patients) over time; colored lines (accompanying slope and p-value) represent linear fit to each disease severity: lm(within-subject Bray-Curtis dissimilarities ~ log(Day, 2), the green shaded area represents the 95% confidence interval of pooled COVID samples); colored boxplots on the right represent within-subject Bray-Curtis dissimilarity by disease severity; green violin plot on the right represents Within-subject Bray-Curtis dissimilarity of all COVID samples. Non-metric multidimensional scaling (NMDS) based on species-level Bray-Curtis dissimilarity matrices from (c) pooled shotgun metagenomes (*n* = 753) and (d) pooled 16S rRNA gene amplicon sequencing samples (*n* = 1,302) with known sampling day; contour plot was generated using the geom_density_2d function in ggplot2 R package, with contour lines colored by day on a log2 scale (see Extended Data Fig. S7 for plots c and d without contour lines). Gut microbiome tended to shift back toward a state that was more similar to healthy controls over time in two representative cohorts (e) PRJNA714459 (shotgun metagenome) and (f) PRJNA703303 (16S amplicon), see Table S3a for microbiome variations of COVID-19 patients at month 0, month 3, month 6, and month 9 to healthy controls (Bray-Curtis dissimilarity, permutations = 999); colored boxplots on the top and on the right represent Bray-Curtis dissimilarity over time in the first and second ordinations respectively. The prevalence of gut microbiome dysbiosis decreases over time from month 0 to month 6 in two representative cohorts (g) PRJNA714459 and (h) PRJNA703303; colored boxplots on the top represent dysbiosis score over time. Temporal shifts in dysbiosis score (median Bray-Curtis dissimilarity of a COVID sample to every healthy control, Methods) of the gut microbiome of COVID-19 patients based on (i) pooled shotgun metagenomes and (j) pooled 16S amplicon samples; blue lines represent loess fit to each disease severity.

Given that gut microbiome dysbiosis appears to be associated with long COVID syndrome,^15,21^ we further characterized the gut microbiome dynamics of patients with COVID-19 in pooled shotgun and 16S data. Although sampling period and frequency were non-uniform across cohorts, the pooled dataset offered a larger sample size for association detection. First, considering only microbiomes in COVID-19 patients that each provided stool samples at multiple time points, we had data from 310 non-Time-zero shotgun metagenomic samples from 165 patients, and 594 non-Time-zero 16S amplicon samples from 387 patients, respectively (Figure 2a,b). To assess the extent of microbiome shift, we calculated within-subject Bray-Curtis dissimilarity of microbiome composition at the species level (Methods). The temporal variation of gut microbiome was substantial, and divergence from the initial baseline (i.e., Time-zero microbiome) over time was much more pronounced for shotgun metagenomic profiles of pooled samples, irrespective of disease severity except for critical COVID-19 patients (Figure 2a; linear regression with log2(day), *p* < .05). This trend was much weaker in 16S amplicon data where significant association was detected only in the category COVID-19 patients without known severity (Figure 2b; linear regression with log2(day), *p* = .013). We then tested if the gut microbiome temporal variation in patients interacted with their microbiome similarity to that of healthy controls. The mean position of the healthy controls in the NMDS ordination plot (based on shotgun metagenomic species-level Bray-Curtis dissimilarity) was very close to the center of contour for the microbiome data from later collection days post infection (Figure 2c), suggesting that the post-infection gut microbiome shifted back to a state more similar to healthy controls over time. This observation was detectable but less pronounced for pooled 16S amplicon profiles compared to that observed for shotgun metagenomic data (Figure 2d). Cohort-dependent variability (e.g., geography, sequencing region of 16S gene) may in part explain the weaker signal in 16S amplicon data.

To investigate these findings in more detail, we next investigated two representative cohorts of Long COVID that provided high numbers of three or four samplings per patient, at month 0, month 3, month 6, and month 9, i.e., PRJNA714459^20^ (*n* = 88 patients, shotgun metagenome) and PRJNA703303^40^ (*n* = 31 patients, 16S amplicon) (Table S1). On the first ordination of data separation for both cohorts, the distance between the microbiome of patients with COVID-19 and that of healthy controls decreased over time (Figure 2e,f). Patients with COVID-19 when admitted to hospital exhibited significantly different gut microbiome compared to that of 6^th^ month in both studies (*p* < .001, PERMANOVA, Bray-Curtis dissimilarity, Table S3a). This trend was further supported by the lower microbiome difference between COVID-19 patients and healthy controls over six months (r^2^ = 0.051 to 0.026 (PRJNA714459)^20^, 0.096 to 0.053 (PRJNA703303)^40^, *p* < .001, PERMANOVA, Bray-Curtis dissimilarity, Table S3a), notwithstanding a higher microbiome distance at the 9^th^ month compared to that of the 6^th^ month in PRJNA714459 cohort.^20^ The small sample size at 9^th^ month (*n* = 9) may account for this greater microbiome dissimilarity (Table S3a). The frequency of microbiomes classified as dysbiotic also decreased over time in both cohorts at the 6^th^ month (Figure 2g,h), consistent with our observations above. Moreover, we measured the gut microbiome variation in patients between acute and post-acute stages in eight cohorts (three shotgun metagenome, five 16S amplicon; Table S3b). Patients with acute COVID-19 exhibited significantly different gut microbiome compared to that of post-acute stage in three cohorts (*p* < .05, PERMANOVA, Bray-Curtis dissimilarity, Table S3b).

To gain further insights into microbial dynamics during the progression of COVID-19 disease severity, we applied local polynomial (loess) regression to determine the temporal variation of dysbiosis score (i.e., median Bray-Curtis dissimilarity of a COVID microbiome to that of every healthy control, Methods) in the gut microbiome of patients with differing disease severity of COVID-19. In most cases with known severity, the dysbiosis score in the gut microbiome increased progressively to a peak during 7–30 days, and then declined (Figure 2i,j), whereas for the COVID-19 patients who died, the dysbiosis score increased over time and beyond the maximum peak time (~ day 73) (Figure 2j). However, this trend was not apparent for the COVID-19 patients with severe symptoms from the metagenomic data and the uncategorized COVID.

### Robust microbiome signature linked to COVID-19 disease severity across cohorts

**Figure 3.**
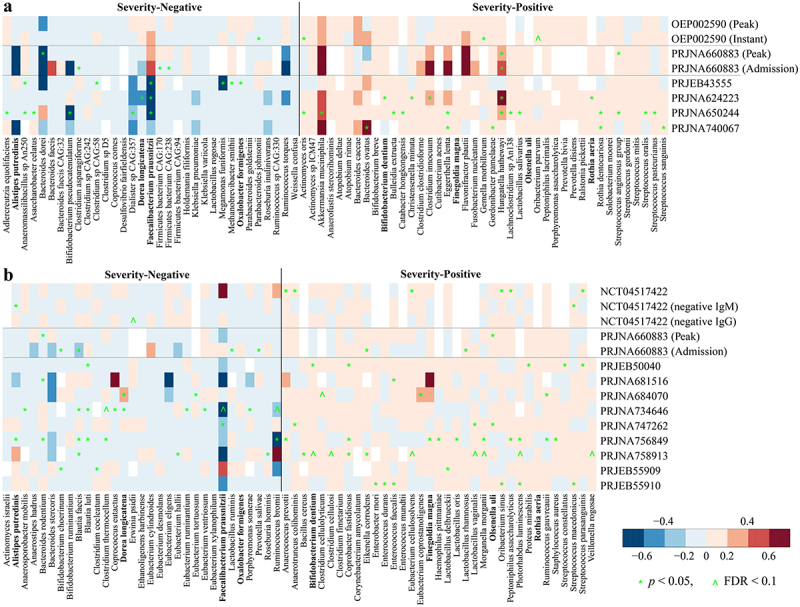
Reproducible microbial species linked to disease severity of COVID-19 across (a) shotgun metagenomic and (b) 16S amplicon cohorts. the color gradient indicates linear regression coefficient of severity (i.e., severity score and/or negative IgM and IgG) for each cohort (computed on relative abundance of first stool sample only from each subject with covariates adjusted); white indicates not detected in the cohort; ^ indicates false discovery rate (FDR) adjusted *p* < .1; * indicates *p* < .05; species names in bold indicate overlapping biomarkers between shotgun metagenomic and 16S amplicon data.

We next sought to identify microbial species differentially associated with COVID-19 disease severity. The disease severity score was derived from Chinese Clinical Guidance for COVID-19 Pneumonia Diagnosis and Treatment, the WHO clinical progression scale, and with reference to anti-covid virus IgM and IgG antibodies whose levels have been reported as informative for diagnosis, severity classification, and clinical management^41^ (please see Methods for details). Covariate-adjusted regression analysis based on shotgun metagenomic data identified 74 microbial species, including 33 “severity-negative” species and 41 “severity-positive” species (Figure 3a). In 16S amplicon data, we identified 31 severity- negative species and 35 severity-positive species (Figure 3b). Two species (*Bacteroides massiliensis* and *Fusobacterium mortiferum*) that had different directions of association between the shotgun and 16S sequencing cohorts were excluded from the downstream analysis. Eight species replicated across shotgun and 16S sequencing cohorts and were associated in the same direction (Figure 3). Of these, *Alistipes putredinis*, *Dorea longicatena*, *Faecalibacterium prausnitzii*, and *Oxalobacter formigenes* were depleted in patients with COVID-19, while *Bifidobacterium dentium*, *Finegoldia magna*, *Olsenella uli*, and *Rothia aeria* were enriched in patients with COVID-19. *F. prausnitzii*, *D. longicatena*, and *A. putredinis* are short chain fatty acid (SCFA) producers in the human gut,^42–44^ and *F. prausnitzii* and *A. putredinis* have been associated negatively with Inflammatory Bowel Disease.^42^ *O. formigenes* plays a critical role in gut oxalate metabolism and calcium oxalate kidney stone disease.^45^ *B. dentium* is an oral pathogen that can cause bloodstream infections.^46^ Oral bacteria, which have been reported as elevated in the gut microbiome in a number of dysbiosis-related diseases, featured in the severity-positive taxa according to both data types, including two species of *Rothia* and several *Streptococcus* spp. Irrespective of sequencing data type, COVID-depleted species are primarily commensals in the human gut, whereas approximately 80% and 63% COVID-enriched metagenomic and 16S species, respectively, have been reported in cases of hospital-acquired bacteremia (Table S4). Recent studies have found bloodstream infections of COVID-enriched species in patients with COVID-19,^47–49^ or increased levels of bacterial DNA in serum,^50^ suggesting impaired barrier function in severe COVID-19 patients that can contribute to secondary bacterial infections.

Compared to the findings of the original studies, six severity-negative metagenomic species (i.e., *Faecalibacterium prausnitzii*, *Bifidobacterium pseudocatenulatum*, *Dorea longicatena*, *Adlercreutzia equolifaciens*, *Coprococcus comes*, and *Dialister* sp. CAG:357) were depleted in COVID in at least two original studies, and for severity-positive metagenomic species, four (i.e., *Bacteroides caccae*, *Bacteroides ovatus*, *Akkermansia muciniphila*, and *Clostridium innocuum*) were enriched in at least two original studies (Table S1). Among three studies based on 16S amplicon data with species-level analysis, six severity-negative species (i.e., *Faecalibacterium prausnitzii*, *Dorea longicatena*, *Alistipes putredinis*, *Blautia faecis*, *Blautia luti*, and *Ruminococcus bromii*) were depleted in COVID and one severity-positive species (i.e., *Enterococcus faecalis*) was enriched in COVID in at least one original study, respectively (Table S1). Of these, *Faecalibacterium prausnitzii* and *Dorea longicatena* replicated in both shotgun and 16S sequencing studies.

### Microbiome signatures for COVID-19 severity category prediction across cohorts

**Figure 4.**
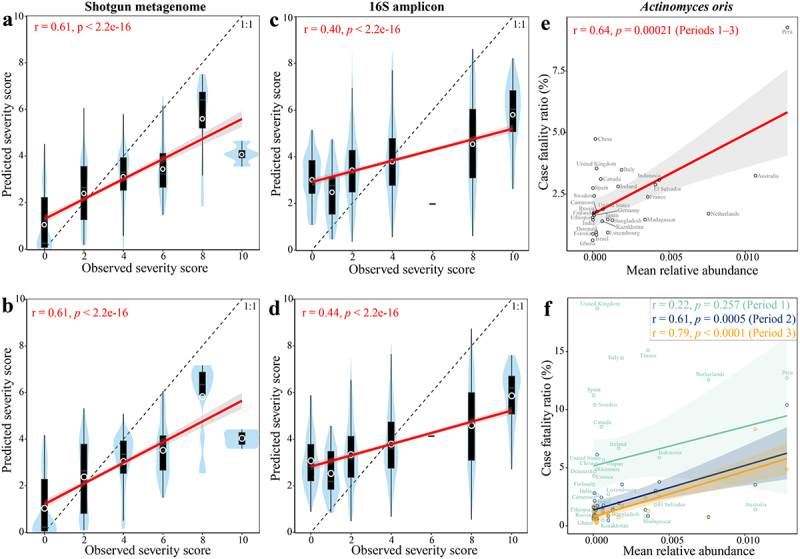
Microbiome signature taxa predict disease severity category of COVID-19 across cohorts. Predicted severity score from random forest (RF) regression model trained on (a) 74 metagenomic biomarkers, (b) all metagenomic species, (c) 66 16S biomarkers, and (d) all 16S species is associated with observed severity score. The mean relative abundance of *Actinomyces oris* (a severity-positive metagenomic biomarker) in the gut of healthy controls was positively associated with mortality rate of COVID-19 across 29 countries that reported at least 1,000 confirmed SARS-CoV-2 cases per country (e) during periods 1–3 and (f) each period. The abundance of gut metagenomic data of healthy controls were obtained from ExperimentHub^51^ and mortality before COVID-19 vaccine administration in each country was obtained from JHU CSSE COVID-19 Dataset (please see Methods for details). r and p in red indicate Pearson’s correlation coefficient and *p* value; label on plot c indicates country. Solid lines show results from simple linear regression (lm function in R), with 95% confidence intervals shown in the shaded areas. The white circle represents the median of predicted severity score; the black bar in the center represents the interquartile range of predicted severity score; the light blue violin plot represents the kernel density of predicted severity score.

To test if the presence of elements in the gut microbiome could predict COVID-19 disease severity category, we predicted the severity score of the remaining hold-out cohort using a random forest (RF) regression model that was trained on the metagenomic and 16S biomarker profiles of all but one cohort (Methods). When we analyzed pooled microbiome datasets from shotgun metagenomic cohorts or 16S amplicon cohorts, microbiome biomarkers could accurately predict severity score (metagenomic biomarker: *r* = 0.61, *p* < .0001, Pearson’s correlation, Figure 4a; 16S biomarker: *r* = 0.40, *p* < .0001, Pearson’s correlation, Figure 4c). Compared to that based on metagenomic biomarkers, the RF regression model based on all the metagenomic species performed to the same accuracy (AUC = 0.77, *p* = 0.87, Wilcoxon rank-sum test, Extended Data Fig. S8a) and the same association between the observed and predicted severity scores (*r* = 0.61, *p* < .0001, Pearson’s correlation, Figure 4b). Although the RF regression model based on all 16S species did not improve the model performance compared to that based on biomarkers (Extended Data Fig. S8a), a slightly stronger association between the observed and predicted severity scores was obtained for all 16S species compared to that of 16S biomarkers (Figure 4d). We found that *Fusicatenibacter saccharivorans* had the highest rank in the model for shotgun data, and *Eubacterium rectale* ranked highest in the 16S data model (Table S5). Other species including *Agathobaculum butyriciproducens*, *Blautia obeum*, *Blautia wexlerae*, *Parabacteroides distasonis*, *Adlercreutzia equolifaciens*, *Roseburia faecis*, *Clostridium innocuum*, *Clostridium sporosphaeroides*, and *Anaerostipes hadrus*, were also crucial to prediction accuracy, with 27 (shotgun model) and 19 (16S model) out of top-ranked species (i.e., 74 for shotgun data and 66 for 16S data) for COVID severity prediction amongst the identified biomarkers, respectively (Extended Data Fig. S8b). Of these top-ranked species among the identified biomarkers, *A. equolifaciens* and *A. hadrus* were overlapping in both shotgun and 16S models (Extended Data Fig. S8b).

Next, we tested if the microbiome biomarkers were associated with population-based outcome of COVID-19, meaning the mortality rate within cohorts. We obtained the abundance of metagenomic biomarkers in the gut of healthy controls from 29 countries from ExperimentHub^51^ and confirmed COVID-19 cases and deaths before COVID-19 vaccine administration (i.e., December 8, 2020) in each country from JHU CSSE COVID-19 Dataset (please see Methods for details). We found that the mean relative abundance of *Actinomyces oris* (i.e., a severity-positive metagenomic biomarker) in the gut of healthy controls was positively associated with the mortality rate of COVID-19 within 29 countries that reported at least 1,000 confirmed SARS-CoV-2 cases per country (*r* = 0.64, FDR = 0.0016, Pearson’s correlation, Figure 4e). However, no significant association was observed for the other metagenomic biomarkers (FDR > 0.1, Pearson’s correlation, Table S6). Considering the time lapse of spread of COVID-19 across countries, we next estimated the association of *A. oris* with mortality rate based on three different periods (i.e. Period 1: 2020.01.22–2020.06.08, Period 2: 2020.06.09–2020.09.08, and Period 3: 2020.09.09–2020.12.08). A positive association was replicated in each period, with statistical significance in Period 2 (*r* = 0.61, *p* = .0005) and Period 3 (*r* = 0.79, *p* < .0001, Pearson’s correlation, Figure 4f). *Actinomyces oris*, formerly known as *A. naeslundii*, is the most abundant *Actinomyces* species in the human oral cavity.^52,53^ *A. oris* plays important roles in biofilm formation through interactions with other bacteria and can cause human actinomycosis lesions.^53^ Overall, these results suggest that a diagnostic test targeting this microbial biomarker in stool could have utility for proactive management of COVID-19 patients and for Long COVID.

### Overlapping microbiome signatures in COVID-19 and IBS

Gut microbiome alteration or dysbiosis in COVID-19 patients may persist even beyond 1-year of recovery.^20,21,25^ GI symptoms that persist six months or longer are increasingly being recognized as important manifestations of Long COVID-19.^12,13^ It has been speculated that COVID-19 infection may trigger irritable bowel syndrome (IBS),^13,54^ i.e. a type of post infectious IBS (PI-IBS), and 5.3%–30.6% of COVID-19 patients developed a new-onset IBS-like disorder that met Rome III or IV criteria.^55–58^ COVID-19 is associated with an increased risk of IBS.^59^ It is well recognized that infection with a variety of microorganisms may act as a trigger for PI-IBS,^60^ and that alteration in the gut microbiome has been associated with some classes of IBS,^61^ but it is unknown if there are any shared microbiome biomarkers for COVID-19 and IBS. To test this, we selected from the literature two IBS case-control studies which satisfied the criteria of identifying gut microbiome taxa differentially abundant between IBS and healthy controls at the species level,^61,62^ and which were based on shotgun metagenomic sequence data. Studies focussing solely on PI-IBS were not available; the two studies did not discriminate between IBS types. Compared to the differentially abundant metagenomic species reported in these two IBS studies,^61,62^ 21 out of the 74 metagenomic biomarkers for COVID (Table S7) were significantly different in abundance between IBS and healthy controls in at least one study (FDR <0.1). Of these 21 differentially abundant species, four species (i.e., *Alistipes putredinis*, *Faecalibacterium prausnitzii*, *Parabacteroides goldsteinii*, and *Parabacteroides johnsonii*) were depleted and fourteen, including *Actinomyces oris*, *Clostridium* spp. (*C. clostridioforme*, *C. innocuum*), *Eggerthella lenta*, *F. plautii*, *Gordonibacter pamelaeae*, *Hungatella hathewayi*, *Rothia dentocariosa*, *Streptococcus* spp. (*S. anginosus* group, *S. gordonii*, *S. mitis*, *S. oralis*, *S. sanguinis*), were in elevated abundance in both COVID-19 and IBS, whereas the directionality of the difference for four species was reversed in IBS compared with COVID-19 (Table S7). Of these species, three species (i.e., *E. lenta*, *S. anginosus* group (SAG), and *S. sanguinis*) overlap in our present study and both IBS studies.^61,62^

### Habitual dietary ingredients are associated with microbiome biomarkers of COVID

**Figure 5.**
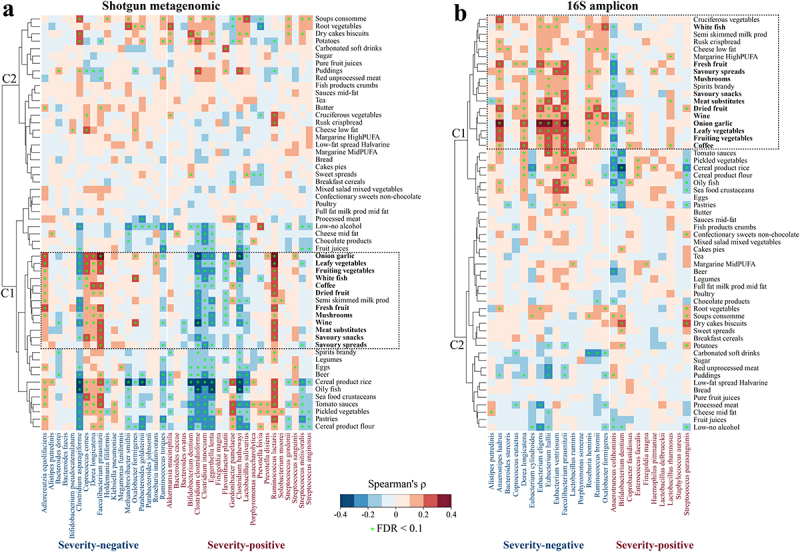
Associations between dietary intake (assessed through food frequency questionnaires and parsed by the NU-AGE food classification system) and gut microbiome biomarkers. Detected (a) shotgun metagenomic and (b) 16S amplicon biomarkers in the healthy individuals of published datasets (see Methods). Cluster (c) was generated with heatmap.2 function using a complete agglomeration method based on Euclidean distance. The color gradient indicates Spearman’s correlation coefficient (ρ) for each cohort; * indicates FDR adjusted *p* < .1.

Microbiome-based therapies against COVID-19 have attracted recent interest^15^ but pose technical challenges, whereas dietary adjustment could be a simple and practical means to ameliorate the dysbiotic gut microbiome. We therefore explored associations between dietary intake and abundance of the identified microbiome biomarkers in published datasets for heathy individuals.^61,63,64^ We identified significant associations between microbial severity biomarkers and diet intake, and the hierarchical correlation analysis divided the foods into self-organizing clusters based on shared correlation patterns with microbiome taxa (Figure 5). For shotgun sequence data (Figure 5a), foods in cluster 1 (see also Extended Data Fig. S9) were potentially beneficial for patients with COVID-19 with respect to effect on microbiome COVID severity markers, although associations were not completely consistent across all marker taxa (e.g., *Clostridium asparagiforme*, *Ruminococcus torques*, and *Ruminococcus lactaris*). 16S analysis identified cluster 1 composed of potentially beneficial dietary ingredients, overlapping with all the potentially beneficial ingredients based on shotgun analysis (Figure 5b). In addition, three other foods in 16S cluster 1 (i.e., cruciferous vegetables, low fat cheese, and spirits) were also potentially beneficial ingredients (Figure 5b). The foods indicated by this approach as being potentially beneficial or at least microbiome-restorative for patients with COVID-19 include vegetables, fruit, meat substitute, savory spreads and snacks, and white fish, as well as alcohol and coffee. The identification of these putatively microbiome-targeting foods provides a basis for designing dietary intervention trials for ameliorating COVID-19 symptoms or promoting recovery.

### Altered gut microbiome proportions of archaea, fungi, viruses, and parasites in COVID patients and their association with disease severity

**Figure 6.**
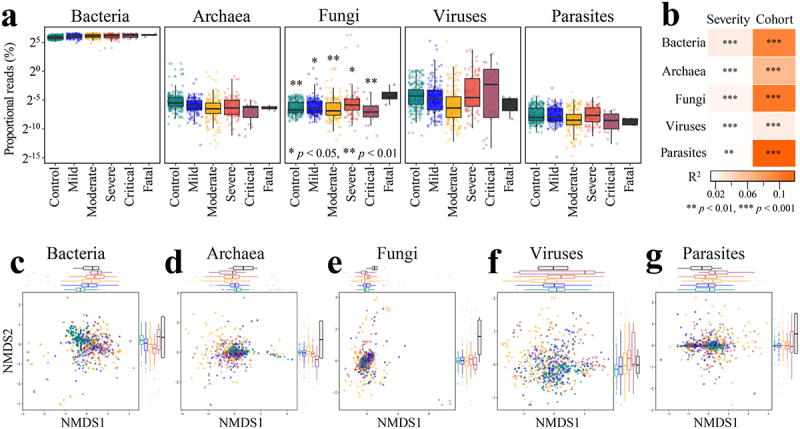
Altered proportions of gut bacterial, archaeal, fungal, viral, and parasitic communities in COVID patients are associated with disease severity. A. Distribution of proportional read (log2 scale) representation of five different microbial kingdoms in the shotgun metagenomes as a function of known disease severity (*n* = 718); *p < .05, **p < .01 (Fatal versus other disease category, Wilcoxon rank-sum test). B. COVID disease severity is significantly associated with differences in the bacterial, archaeal, fungal, viral, and parasitic community proportions across cohorts (first stool sample only from each subject, *n* = 537); microbiome variation was measured by PERMANOVA based on Bray-Curtis dissimilarity matrices computed on relative abundance with cohort adjusted and marginal sums of squares applied, i.e., adonis2(relative abundance matrix ~ severity + cohort, permutations=999, method=“bray”, by=“margin”). Non-metric multidimensional scaling (NMDS) of microbiome-severity correlation based on species-level Bray-Curtis dissimilarity of pooled shotgun metagenomes with known disease severity (*n* =718) for (c) bacteria, (d) archaea, (e) fungi, (f) viruses, and (g) parasites; colored boxplots on the top and the right represent Bray-Curtis dissimilarity by disease severity in the first and second ordinations, respectively.

The human gut is a complex ecosystem, harboring microorganisms from several kingdoms (primarily bacteria, but also archaea, fungi, protists, and viruses). Several recent studies reported alterations in the community composition for gut fungi and viruses in COVID-19.^65–69^ However, it was unclear whether the gut community proportions or compositions of non-bacterial kingdoms were associated with disease severity of COVID-19 across cohorts. On average, 67.3% of the total sequences were annotated from the Kraken2 pipeline (see Methods), with 66.5% as bacteria, 0.05% as archaea, 0.30% as fungi, 0.41% as viruses, and 0.007% as parasites (Figure 6a). The proportion of reads assigned to fungi from the microbiome of subjects in the fatal outcome group was significantly higher than all other disease groups (*p* < .05, Wilcoxon rank-sum test, Figure 6a). However, the fatal group only consists of four samples that were from the US cohort (i.e., PRJNA660883).^70^ The maximum fungal read proportion in healthy controls was 0.06%, whereas fungi dominated two samples of COVID-19 subjects with severe disease (Figure 6a, Extended Data Fig. S10). The fungal read proportion of these two subjects declined over time (near 80% on day 1, > 17% on day 5, < 0.01% on day 23). One species *Candida glabrata* dominated the fungal community (Extended Data Fig. S10), suggesting that an invasive infection of *Candida glabrata* had occurred, an event previously reported as an upper respiratory infection in one COVID-19 patient.^71^ The maximum viral read proportion in healthy controls was 1.2%, whereas the maximum viral read proportion in patients with COVID-19 was 38.7%. Relative to the maximum viral read proportion in healthy controls, 32 metagenomes of COVID-19 patients had higher viral sequence loads, with eight abundant phages detected (>1% in at least one metagenome), including seven crAssphages (i.e., Gut phage BED-2012, crAssphage cr112_1, crAssphage cr109_1, crAssphage cr10_1, crAssphage cr113_1, crAssphage cr6_1, and *Bacteroides* phage crAss001) and the *Klebsiella* virus K244. COVID-19 disease severity was associated with the gut bacterial, archaeal, fungal, viral, and parasitic community compositions, accounting for 1.4%–2.5% of the microbiome variation while adjusting for cohort (*p* < .01, PERMANOVA, Bray-Curtis dissimilarity, Figure 6b–g). However, we note that over 30% of the total sequence reads were not annotated. This may constitute a potential bias in characterizing the proportional variation by severity of the non-bacterial gut communities. Nevertheless, these findings suggest the likelihood of significant variations in gut proportions of non-bacterial kingdoms in COVID-19 and indicate the need for scrutiny of these communities in future studies.

## Discussion

### Temporal dynamics of gut microbiome alterations in COVID-19

Individual studies have consistently demonstrated that gut microbiome alterations are associated with COVID-19 disease,^21,22,24,26^ but it is unclear whether this alteration or dysbiosis contributes to COVID-19 disease severity, if COVID-19 disease processes drive changes in microbiota composition, or if microbiota differences are unrelated to COVID-19 itself but reflect other coincidental demographic, disease, or dietary factors. Here, we confirmed robust severity-associated alterations in microbiome composition across multiple COVID-19 cohorts. A previous meta-analysis found that SARS-CoV-2 viral load in the upper respiratory tract peaked in the first week of infection, and no live virus was detected beyond day 9 of infection.^72^ However, extended shedding of SARS-CoV-2 RNA occurs in the gut for some people.^73,74^ In the current microbiome meta-analysis, gut microbiome dysbiosis peaked after the viral load peak, i.e., during days 7–30 (Figure 2i,j), suggesting that the altered gut microbiome is primarily a consequence of SARS-CoV-2 infection. Mechanistically, this could be related to multiple factors including elevated immune cell activity, but exacerbated by external factors during treatment such as altered dietary intake, use of medications, and change in environment (e.g. hospital setting).

Recent studies have suggested that gut microbiome dysbiosis in patients with COVID-19 was related to an increased risk of developing bacteremia,^75^ which was further evidenced in a recent mouse experiment.^76^ Among the identified severity-positive species (Figure 3), 80.5% (33/41) of metagenomic biomarkers and 62.9% (22/35) of 16S biomarkers have been reported in cases of hospital-acquired bacteremia, respectively (Table S4). Of these, nine species, including *Actinomyces oris*, *Bacteroides ovatus*, *Enterococcus faecalis*, *Morganella morganii*, *Prevotella bivia*, *Rothia aeria*, *Staphylococcus aureus*, SAG, and *Streptococcus sanguinis*, have been found in bloodstreams of COVID-19 patients. In addition to bacterial infection in the bloodstream, fungal bloodstream infections have also occurred in patients with COVID-19.^68^ *Aspergillus*, *Candida* and *Mucorales* species were the most common pathogens that cause fungal coinfections in COVID-19.^68^ The predominant sequences in the gut of two COVID-19 subjects with severe disease were represented by *Candida glabrata* (>75% on day 1, Extended Data Fig. S10), which has been reported in cases of fungal bloodstream infections in patients with COVID-19.^77,78^ However, the mere identification of severity-associated taxa which are sometimes found in bacteremia elsewhere does not prove causality, and further investigation is therefore warranted.

### Predictive microbiome markers of disease severity category

The identification of reproducible microbiome biomarkers for COVID-19 disease severity could help develop microbiome-based surveillance diagnostics and for stratifying patients to antiviral therapies. We developed machine learning models capable of predicting severity category of patients with COVID-19. The RF regression model trained on pooled cohort data using microbiome biomarkers was predictive of disease severity category. However, one limitation is the criteria to define the disease severity. Although not exactly same across all cohorts, the primary severity criteria were Chinese Clinical Guidance for COVID-19 Pneumonia Diagnosis and Treatment, and WHO criteria, which are similar to each other (see Table S1 for Severity Criteria and Descriptor of individual cohorts). These results suggest that the RF models are globally applicable, highlighting that these COVID severity-associated microbial biomarkers may be used to predict disease severity category anywhere in the world. It will be interesting to assess in future studies if these same biomarkers can predict disease severity category in other severe infectious disorders.

Although less than 14% of the identified disease severity-associated biomarkers replicated in the original studies (Table S1), this meta-analysis provides generalizable microbial signatures associated with disease severity. For the meta-analysis, the association of biomarkers with disease severity does not need to be significant for every cohort, but it does highlight the consistent trends across cohorts.

### Stronger severity associations in metagenomic data than 16S amplicon profiles

In general, the data interactions we observed were more pronounced in shotgun metagenomic data than 16S amplicon data, e.g., disease severity-associated dysbiosis (Figure 1e–g, Extended Data Fig. S4c), temporal variation of gut microbiome of individuals (Figure 2b,d), and severity category prediction accuracy (Figure 4a–d). The highly variable copy number of 16S rRNA gene across bacterial taxa^79^ and inherent technical differences^80^ (i.e., different levels of sequence variation of the 16S rRNA gene hypervariable regions in different taxa) across 16S cohorts (Table S1) may explain the lower effect size observed in 16S cohorts. Other technical variations, e.g., sample collection, DNA extraction, sequencing platform, sequencing depth, and annotation pipelines may also affect the taxonomic profiling. Like our recent meta-analysis,^81^ we used MetaPhlAn 3^33^ and SPINGO^34^ to annotate metagenomic and 16S species-level taxonomic profiling, respectively, because they provide highest accuracy at the (sub)species level.^82,83^ MetaPhlAn, computationally efficient, has been applied to process human microbiome data and develop the ExperimentHub,^51^ including over 20 thousands of annotated microbial metagenomes from human so far. Annotation from MetaPhlAn will facilitate the generalization of the impact and scientific relevance of research findings. Seven of the eight shotgun metagenomic cohorts studied were from China, whereas the 16S amplicon cohorts were much more heterogeneous for geography and ethnicity, which may also contribute to the weaker associations for 16S data. To achieve high taxonomic resolution, shotgun metagenomic sequencing is recommended, but it is still relatively costly. Shallow shotgun metagenomic sequencing with as low as 0.5 million sequences per sample seems to be a cost-effective alternative to 16S sequencing with improved taxonomic resolution,^84^ and may be a useful clinical adjunct for COVID-19 studies. Only eight microbiome biomarkers replicated across shotgun metagenomic and 16S sequencing cohorts through covariate adjusted regression (Figure 3). A limitation of the covariate adjusted regression analysis stemmed from the variation of metadata quality across cohorts and lack of important metadata (e.g., sex, age, medication, and comorbidities) in certain cohorts may influence the results. The reasons mentioned above for the weaker associations for 16S data could also contribute to the limited overlapping microbiome signatures.

### Altered microbiome and COVID sequelae: COVID as a trigger of IBS?

Alteration or dysbiosis in the gut microbiome has been implicated in both COVID-19^20,21,25^ and IBS,^61^ and recent studies have suggested that COVID-19 infection may trigger PI-IBS.^55–58^ By comparing COVID-19 biomarkers to the differentially abundant species between IBS and healthy controls in published IBS case – control studies,^61,62^ we found that, of the 74 metagenomic species, 18 were significantly enriched or depleted in both COVID-19 and IBS in at least one study. *E. lenta*, SAG, and *S. sanguinis*, disease-enriched species, overlap in both the present study and two IBS studies.^61,62^ *E. lenta*, an emerging pathogen, can cause bloodstream infections and is associated with multiple diseases.^85–87^ The most common diseases associated with *E. lenta* bacteremia include cancer, diabetes, and cardiovascular diseases,^88^ and the main sources of *E. lenta* bacteremia were associated with intra-abdominal infections.^87–89^ *Eggerthella* bacteremia was recently reported in a patient with COVID-19.^90^ SAG is currently being found to cause invasive infections at almost every body site.^91–93^ Bloodstream infections of SAG have been reported in patients with COVID-19.^94,95^ SAG-bacteremia has been linked to worse disease outcomes in patients,^91,92^ which may predispose to worse outcomes of COVID-19. *S. sanguinis*, an oral commensal, is one of the most common causes of infective endocarditis.^96^ Recent evidence suggests that infective endocarditis caused by *S. gordonii* that belongs to the *S. sanguinis* group is associated with COVID-19 infection.^97^ *A. oris* associated with mortality rate of COVID-19 (Figure 4e,f) was also enriched in patients with IBS.^62^ Although persistent GI symptoms are common in COVID-19 survivors after six months of infection,^12,13^ almost a quarter of microbiome biomarkers for COVID-19 replicate in IBS, suggesting that the overlapping gut microbiome signatures are likely associated with the development of IBS, or with an inflammatory state characteristic of IBS.

### Potential dietary modulation of gut microbiota for managing long COVID

Pilot studies using fecal microbiota transplantation suggest that targeting the gut microbiome in Long COVID patients might be a promising therapeutic strategy.^98–100^ Our findings highlight that the consumption of plant-based foods, savory spreads and white fish could help ameliorate the dysbiotic gut microbiota in patients with COVID-19 and Long COVID (Figure 6). Interestingly, we found that consumption of alcohol may be also helpful in gut microbiome recovery (Figure 6, Extended Data Fig. S9). Consumption of alcohol was associated with increased microbiome diversity in the gut as described previously.^101,102^ Patients with ulcerative colitis consumed less alcohol relative to healthy controls.^103^ However, binge drinking has pathophysiological consequences.^104^ Taken together, these identified foods provide a basis for dietary trial design. Further clinical studies involving patients with Long COVID are needed to assess the clinical efficacy.

### Study limitations

The combined longitudinal analysis in the current study is limited by assembling disparate datasets which may impact the interpretation of the results. The assembled data provide larger sample size for association detection, but there may be issues when using assembled data from patients with different sampling days (1–274), sampling frequency (days to months), and sample size (1–12). The initial dysbiosis score can be high in some patients (Figure 2i), which may be attributed to the baseline difference predating infection or inaccurate infection time data due to delayed diagnosis of COVID-19. Nevertheless, assembling microbiome datasets from disparate cohorts allowed us to characterize the gut microbiome dynamics in patients with COVID-19 more comprehensively over time.

Medications (e.g., antivirals, antibiotics) also explained the observed microbiome associations in several cohorts (Figure 1d), and another limitation is the lack of COVID therapy records in certain cohorts. Also, a recent study evaluated the association between gut microbiome and SARS-CoV-2 vaccines,^105^ and thus the vaccination status in certain patients may have impacted upon identifying consistent microbial signatures across cohorts. Additionally, emerging SARS-CoV-2 variants were associated with an evolution of symptoms,^106^ and further studies are warranted to evaluate the variation of microbiome across people infected with different variants.

With respect to diet-microbiome analysis, the data were from an Irish population, and some biomarkers identified were not detected in the gut microbiome profiles of certain datasets.^61,63,64^ However, in general, these findings are consistent with observed dietary patterns that have the potential to prevent gut inflammation through the gut microbiome,^107^ e.g., consumption of vegetables, fruit, fish, and cereals is associated with a lower abundance of opportunistic pathogen but a higher abundance of SCFA-producers.

### Concluding remarks

Collectively, our current findings support the association of COVID-19 disease severity with consequential gut microbiome alteration/dysbiosis and support the potential of microbiome-based diagnostics for monitoring COVID-19 and disease severity. These biomarkers may also have utility for identifying patients who would most benefit from the available SARS CoV-2 therapeutics. Given the association of the implicated taxa with other diseases with inflammatory components, it is logical to deduce that this altered microbiome, though consequential of the initial infection, contributes to Long COVID. Considering that over 676 million people have been infected globally, there is a need for non-pharmaceutical ways for managing Long COVID, potentially aided by gut microbiota modulation. Dietary intervention studies are warranted to evaluate the clinical efficacy. Further research is also warranted to investigate if the variations in the total proportions and compositions of the non-bacterial microbiome components contribute to acute phase symptomology or to Long COVID. Future efforts should particularly focus on eukaryotic microorganisms that are more prevalent in developing countries where COVID variants are likely to arise, especially focusing on their complex microbiome interactions and roles in COVID-19 progression.

## Methods

### Cohort inclusion and data acquisition

We used PubMed and NCBI to search for studies that published gut shotgun metagenomic data and 16S rRNA amplicon sequencing (16S) data of patients with COVID-19. We curated eight shotgun metagenomic studies and 25 16S cohorts of COVID-19 by April 2022 (Table S1). One 16S cohort (PRJNA728736) with less than 10 microbiome samples from COVID-19 patients was excluded from the downstream analysis. Of the remaining 16S cohorts, one cohort (PRJNA705797) without publicly available metadata for differentiating healthy controls from patients with COVID-19 was excluded as we were unsuccessful in obtaining the information from the authors (Table S1). All sequencing data were downloaded from online repositories (e.g., EBI, NCBI, NODE, ZENODO) (Table S1). We manually curated metadata tables across cohorts. In studies with two control groups (i.e., healthy control and non-COVID-19-related phenomena), we only included the healthy control for comparison. In studies without healthy control, we compared to the healthy controls from all other shotgun metagenomic or 16S studies (i.e., global healthy control) for the dysbiosis analysis. In the PRJNA660302 study,^108^ the subjects with suspected COVID-19 were excluded. In the French cohort (PRJNA787810),^109^ samples from healthcare workers were excluded (except one person who tested positive in stool), because it is unclear whether they had COVID-19 infections. Considering more cohorts could be available after we performed the meta-analysis on the eight shotgun metagenomic and 23 16S amplicon cohorts, we searched in August 2022 to locate more recently released cohorts. We identified nine additional cohorts, including six shotgun metagenomic and four 16S amplicon cohorts (Table S1). Among these cohorts, none of these cohorts has available disease severity of COVID-19 patients, and thus none of these cohorts was included for external validation. 16S samples with less than 3,000 reads and shotgun metagenomic samples with less than 0.5 million reads were excluded from the downstream analysis as suggested by Hillmann *et al*.,^84^ resulting in total of 1,023 shotgun metagenomic and 2,415 16S amplicon samples.

Among all the investigated cohorts, only two 16S cohorts (i.e., PRJNA756849^24^ and NCT04517422^110^ were accompanied by severity score. For the remaining cohorts, each disease severity was assigned to a severity score based on the WHO clinical progression scale,^111^ i.e., 0 for healthy control, 1 for asymptomatic, 2 for mild, 4 for moderate, 6 for severe, 8 for critical, and 10 for fatal, with oxygenation requirement being considered for the severe disease in one cohort (OEP002590)^26^ where extracorporeal membrane oxygenation and tracheal intubation were available. For cohort PRJNA639286,^112^ all patients had “mild or moderate” illness that was not discriminable, and we replaced “mild or moderate” with “moderate” for consistency. For the two cohorts (i.e., OEP002590^26^ and PRJNA660883^70^ that had both peak severity and admission (or instant) severity, we used peak severity in all the analyses and both severities for the identification of microbiome biomarkers. In the PRJNA650244 cohort,^21^ the sampling day is the approximate day estimated from “stool days since negative qPCR”.

### Taxonomic and functional profiling and data preprocessing

Shotgun metagenomes were quality-filtered using TrimGalore v0.6.7 with default settings under the ‘–paired’ option^113^ and were further filtered to remove human and PhiX sequences using Kraken 2^114^ with the human and PhiX genome database.^115^ The decontaminated sequences were reprocessed using MetaPhlAn 3^33^ for species-level taxonomic profiling and HUMAnN 3^33^ for functional profiling (i.e., abundance profiles of UniRef90s gene families). In addition, the decontaminated sequences were reprocessed using Kraken 2^114^ for species-level taxonomic profiling of five microbial kingdoms against the constructed database using all NCBI genomes from five kingdoms (i.e., bacteria, archaea, fungi, viruses, and parasites) on February 18, 2022. The normal human gut is dominated by bacteria, thus limiting the application of de novo assembler-based approach for microbial communities of other kingdoms with very small proportional sequences, whereas Kraken2 method allows to estimate the proportions of these kingdoms in a metagenome. The gene family profiles were further regrouped to KEGG Orthologs (KOs),^35^ Enzyme Commission number (EC number), MetaCyc pathway (Pathway),^36^ and carbohydrate active enzymes (CAZymes).^37^ 16S amplicon sequences were also quality-filtered using Trim Galore with the ‘–paired’ option for paired-end sequences and default settings for single-direction sequences^113^ and then reprocessed using SPINGO^34^ for taxonomic profiling at the species level, with paired-end sequences being concatenated as suggested by Dacey and Chain.^116^

### Statistical analyses

All data analyses were carried out in R 3.6.2 (https://www.r-project.org/). Nonmetric multidimensional scaling (NMDS) was performed for the ordination of microbiome samples with Bray-Curtis dissimilarity based on relative species abundances (Figure 1b,c). The envfit function in vegan^117^ R package was used to fit the variables (e.g., disease severity, cohort) to the two top ordinations. The permutational multivariate analysis of variance (PERMANOVA) was performed with Bray-Curtis dissimilarity based on relative abundance and Aitchison dissimilarity based on centered-log-ratio-transformed (CLR) absolute abundance at the species level for taxonomy and at the metagenome level for functional potential (i.e., copies per million), respectively, through vegan adonis2 function. In the case of longitudinal sample collection, only the first sample from each subject was used in the PERMANOVA (Figure 1d). In addition to disease severity, all other available factors within individual cohorts were included in the model, and the marginal sums of squares was applied to avoid issues related to the order of factors^118:^ adonis2(relative abundance matrix ~ severity + other factors, permutations = 999, method=“bray”, by=“margin”) or adonis2(CLR absolute abundance matrix ~ severity + other factors, permutations = 999, method=“euclidean”, by=“margin”). All the figures were generated in R using gplots package for heatmap and ggplot2 package for other figures.

### Definition of microbiome dysbiosis and within-subject Bray-Curtis dissimilarity

To identify gut samples with highly divergent microbiome compositions, we evaluated the dysbiosis score, i.e., the median Bray-Curtis dissimilarity of a given sample to the healthy controls. Samples beyond the 90^th^ percentile of the distribution in healthy controls were classified as ‘dysbiotic’ in accordance with the classification procedure.^38^ For individual cohorts, dysbiosis score was estimated by comparing to global control datasets in the case of cohort studies that lacked healthy controls (Extended Data Fig. S4c, Extended Data Fig. S5, Extended Data Fig. S6). To assess the extent of microbiome shift over time, we calculated the within-subject dissimilarity, i.e., the Bray-Curtis dissimilarity of species-level microbiome composition of a stool sample collected at one time point to the first collected sample of individual subject.

### Severity-microbiome association analysis

To identify the microbiome biomarkers for COVID-19, a covariate-adjusted regression model was applied to estimate the association between species relative abundance and disease severity score and inflammatory markers reflective of COVID-19 disease severity (i.e., IgM and IgG antibodies) with significant covariates (*p* < .05, PERMANOVA, Bray-Curtis dissimilarity, Figure 1d). If a factor was significantly associated with the microbiota composition in a cohort, it was included in the regression model for each cohort when available. The species that detected in less than four cohorts for shotgun metagenomic data and eight cohorts for 16S amplicon data were excluded in the downstream analysis. We considered a species to be consistently associated with COVID-19 if it was significantly associated (FDR <0.1 or *p* < .05) with the disease severity in at least two cohorts (one of which with FDR < 0.1) and in the same direction in at least 60% cohorts; if it was significantly associated (FDR <0.1) with the disease severity in one cohort and in the same direction in at least 70% cohorts; if it was significantly associated (*p* < .05) with the disease severity in one or two cohorts and in the same direction in at least 70% cohorts; and if it was not significantly associated (*p* > .05) with the disease severity in any cohort and in the same direction in at least 80% cohorts. In the case of contradicting significant associations of species abundance with severity scores (admission, instant, or peak) within a single cohort, the cohort was then dropped out of counting.

### Diet-microbiome association analysis

To identify potentially beneficial diets, we used nonparametric Spearman correlation to test associations between dietary intake and relative abundance of the identified microbiome biomarkers with published datasets that contained both food frequency questionnaire (FFQ) and relative species abundance of gut microbiome for heathy individuals.^61,63,64^ Of the identified biomarkers, 39 shotgun metagenomic species and 21 16S amplicon species were detected in the published datasets. The association was explored for specific food items and aggregated food groups based on the European food classification system. The foods that were positively associated with COVID-depleted microbial biomarkers and negatively associated with COVID-enriched microbial biomarkers were considered as potentially beneficial diets. The false discovery rate (FDR) adjusted p-value less than 0.1 was considered significant.

### Random forest regression model

Random forest (RF) regression model building and evaluation were performed using random forest R package.^119^ RF regression model was trained on all but one cohort, and the prediction accuracy was externally estimated on the remaining hold-out cohort using the relative abundance of the microbiome biomarkers (i.e., 74 shotgun microbiome species or 66 16S amplicon microbiome species), respectively. The comparison of predicted severity score values from RF regression model to the observed severity score values was performed using the Pearson’s correlation for shotgun metagenomic data and 16S amplicon profiles, respectively (Figure 4a–e).

### Association between metagenomic biomarkers and COVID-19 outcome

To identify if gut microbiome biomarkers are associated with population-based outcome of COVID-19, we obtained the gut metagenomic data of healthy controls from ExperimentHub^51^ and confirmed COVID-19 cases and deaths before COVID-19 vaccine administration (i.e., December 8, 2020) in each country from JHU CSSE COVID-19 Dataset (https://github.com/CSSEGISandData/COVID-19/blob/master/csse_covid_19_data/csse_covid_19_daily_reports/12-08-2020.csv). All the metagenomes with less than 0.5 million reads were discarded, and the countries that reported less than 1,000 confirmed SARS-CoV-2 cases per country by December 8, 2020 were excluded. Totally, 12370 gut metagenomes from 29 countries, including Australia, Bangladesh, Cameroon, Canada, China, Denmark, El Salvador, Estonia, Ethiopia, Finland, France, Germany, Ghana, India, Indonesia, Ireland, Israel, Italy, Japan, Kazakhstan, Luxembourg, Madagascar, Netherlands, Peru, Russia, Spain, Sweden, United Kingdom, and United States were included. As the microbiome data from ExperimentHub^51^ were based on shotgun metagenomes, we associated the mean relative abundance of shotgun metagenomic biomarkers in the gut of healthy controls with the mortality rate of COVID-19 within 29 countries using Pearson’s correlation (Table S6). Considering the time lapse of spread of COVID-19 across countries, we further associated *A. oris* with period-based mortality rate (i.e. Period 1: 2020.01.22–2020.06.08, Period 2: 2020.06.09–2020.09.08, and Period 3: 2020.09.09–2020.12.08), which was estimated using the newly confirmed COVID-19 cases and deaths within each period using Pearson’s correlation.

## Supplementary Material

Supplemental Material

Supplemental Material

## Data Availability

The raw sequencing data are available from public sources as identified in Table S1.
