# Supplementary material for: Robust cross-cohort gut microbiome associations with COVID-19 severity
Source: Gut Microbes. 2023 Aug 7;15(1):2242615. doi: 10.1080/19490976.2023.2242615 (PMC10411309; doi:10.1080/19490976.2023.2242615)
Supplement: Supplemental Material [file KGMI_A_2242615_SM9740.docx]

# Robust cross-cohort gut microbiome associations with COVID-19 severity

1. Junhui Li^1,2^, Tarini Shankar Ghosh^1,2^, Rachel McCann^3^, Patrick Mallon^3^, Colin Hill^1,2^, Lorraine
2. Draper^1,2^, David Schult^4^, Liam J. Fanning^5^, Robert Shannon^6^, Corinna Sadlier^5,6^, Mary Horgan^5,6^, Liam
3. O’Mahony^1,2,5^ and Paul W. O’Toole^1,2*^. 5

# 6 Extended Data Figure List

7

# Extended Data Fig. 1. Non-metric multidimensional scaling (NMDS) based on species-level Bray-

1. **Curtis dissimilarity**. (a) pooled shotgun metagenome datasets (n =718) and (b) 16S rRNA gene
2. amplicon sequencing datasets (n = 1,698) from subjects with known COVID disease severity (See
3. Extended Data Fig. 2 and 3 for individual cohorts, respectively). Colored boxplots on the top and the
4. right represent Bray-Curtis distance by disease severity in the first and second ordinations respectively.

# Extended Data Fig. 2. Non-metric multidimensional scaling (NMDS) based on species-level Bray-

1. **Curtis dissimilarity of eight individual shotgun metagenome cohorts.**

# Extended Data Fig. 3. Non-metric multidimensional scaling (NMDS) based on species-level Bray-

1. **Curtis dissimilarity of 20 individual 16S amplicon cohort studies with different COVID disease**

# severity within cohort.

1. **Extended Data Fig. 4**. **Global distribution of microbial dysbiosis scores as a measure of disease**
2. **activity.** Density of microbial dysbiosis scores of (a) Shotgun metagenomes (n = 1,023) and (b) 16S
3. rRNA gene amplicon sequencing samples (n=2,415); (c) Microbiome dysbiosis frequency is associated
4. with disease severity. For cohorts without healthy controls, microbiome dysbiosis was estimated by
5. comparing with the pooled data from healthy controls.

# Extended Data Fig. 5. Distribution of microbial dysbiosis scores as a measure of disease activity

1. **in individual shotgun metagenomic cohorts.** The dysbiosis score of (f) PRJNA660883 cohort was
2. compared with that of 270 global healthy controls (i.e., healthy controls from all other cohorts) as there
3. was no healthy controls in this cohort.

# Extended Data Fig. 6. Distribution of microbial dysbiosis scores as a measure of disease activity

1. **in individual 16S amplicon cohorts.** Dysbiosis scores of (a) NCT04517422, (e) PRJNA660883, (j)
2. PRJNA734646, (m) PRJNA747262, (r) PRJEB50040, (s) Dublin Ireland, (t) Cork Ireland, (u)
3. PRJNA787810, (v) PRJNA818796, and (w) PRJNA639286 cohorts were compared with 413 global
4. healthy controls (i.e., healthy controls from all other cohorts) as there was no healthy control in these
5. cohorts.
6. **Extended Data Fig. 7**. Non-metric multidimensional scaling (NMDS) based on species-level Bray-
7. Curtis dissimilarity matrices from (**a**) pooled shotgun metagenomes (n = 753) and (**b**) pooled 16S
8. rRNA gene amplicon sequencing samples (n = 1,302) with known sampling day.

# Extended Data Fig. 8. Predictive accuracy of a random forest (RF) regression model based on

1. **biomarkers was comparable to that based on all species, irrespective of shotgun metagenomic or**
2. **16S amplicon data.** a. Predictive accuracy resulting from leave-one-out cross validation as measured
3. by the area under the curves (AUCs). b. Number of cohorts where the top important features (74 for
4. metagenomic data and 66 for 16S data) of the RF regression model overlapped with biomarkers
5. identified in Fig. 3.

# Extended Data Fig. 9. Associations between dietary intake (assessed through food frequency

1. **questionnaires and parsed by the European food classification system) and gut microbiome**
2. **biomarkers**. Detected (a) shotgun metagenomic and (b) 16S amplicon biomarkers in the healthy
3. individuals of published datasets (see Methods). Cluster (C) was generated with heatmap.2 function
4. using a complete agglomeration method based on Euclidean distance. The color gradient indicates
5. Spearman's correlation coefficient (ρ) for each cohort; * indicates FDR adjusted p < 0.1.

# Extended Data Fig. 10. Fungal read proportion in the gut metagenome of two subjects over time.

1.
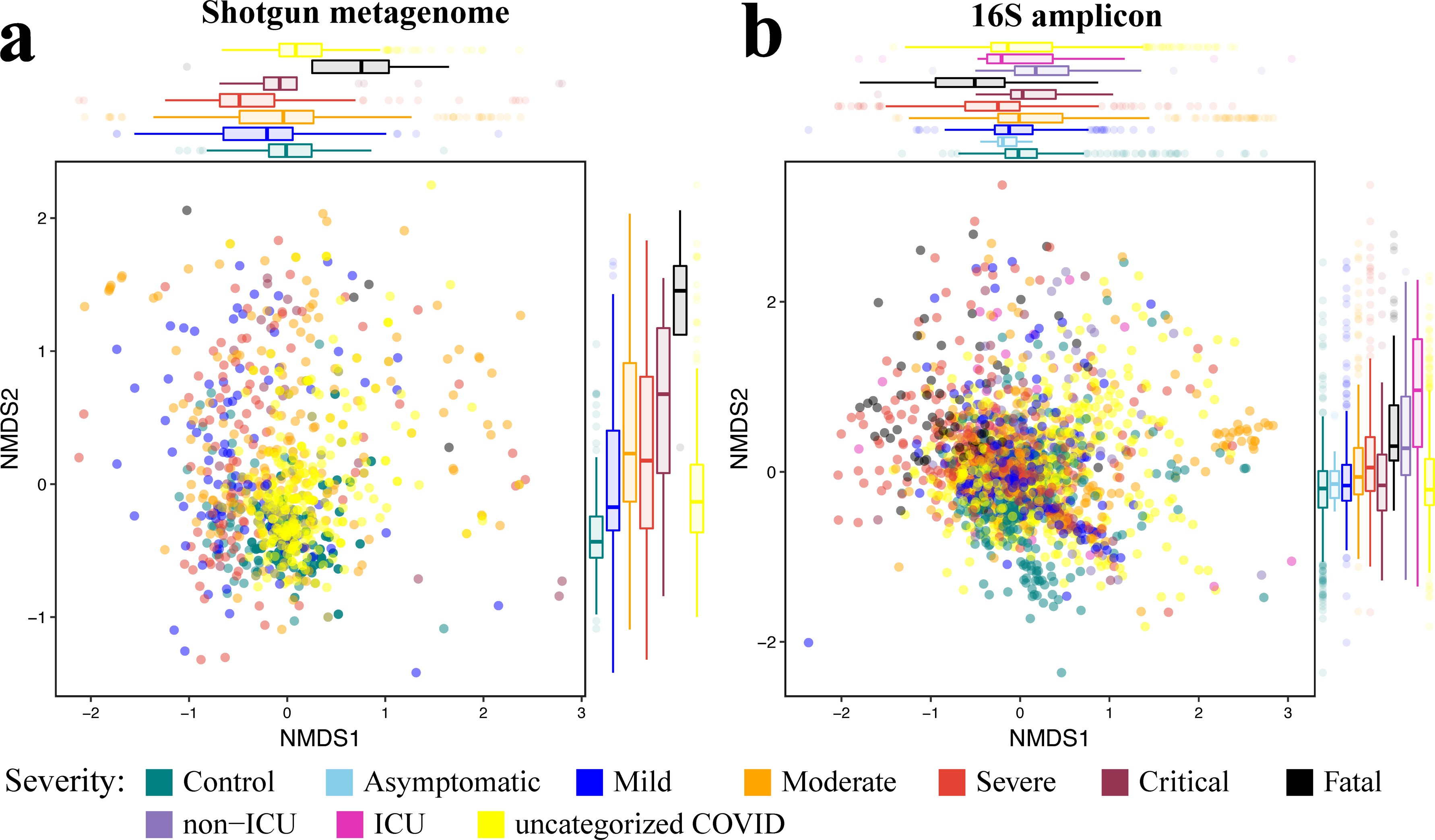
Color indicates days since diagnosis. 50

51

52

# Extended Data Fig. 1. Non-metric multidimensional scaling (NMDS) based on species-level Bray-

1. **Curtis dissimilarity**. (a) pooled shotgun metagenomes (n =718) and (b) pooled 16S rRNA gene
2. amplicon sequencing samples (n = 1,698) with known disease severity (See Extended Data Fig. 2 and 3
3. for individual cohorts, respectively). Colored boxplots on the top and the right represent Bray-Curtis
4. distance by disease severity in the first and second ordinations respectively. 58

59

60


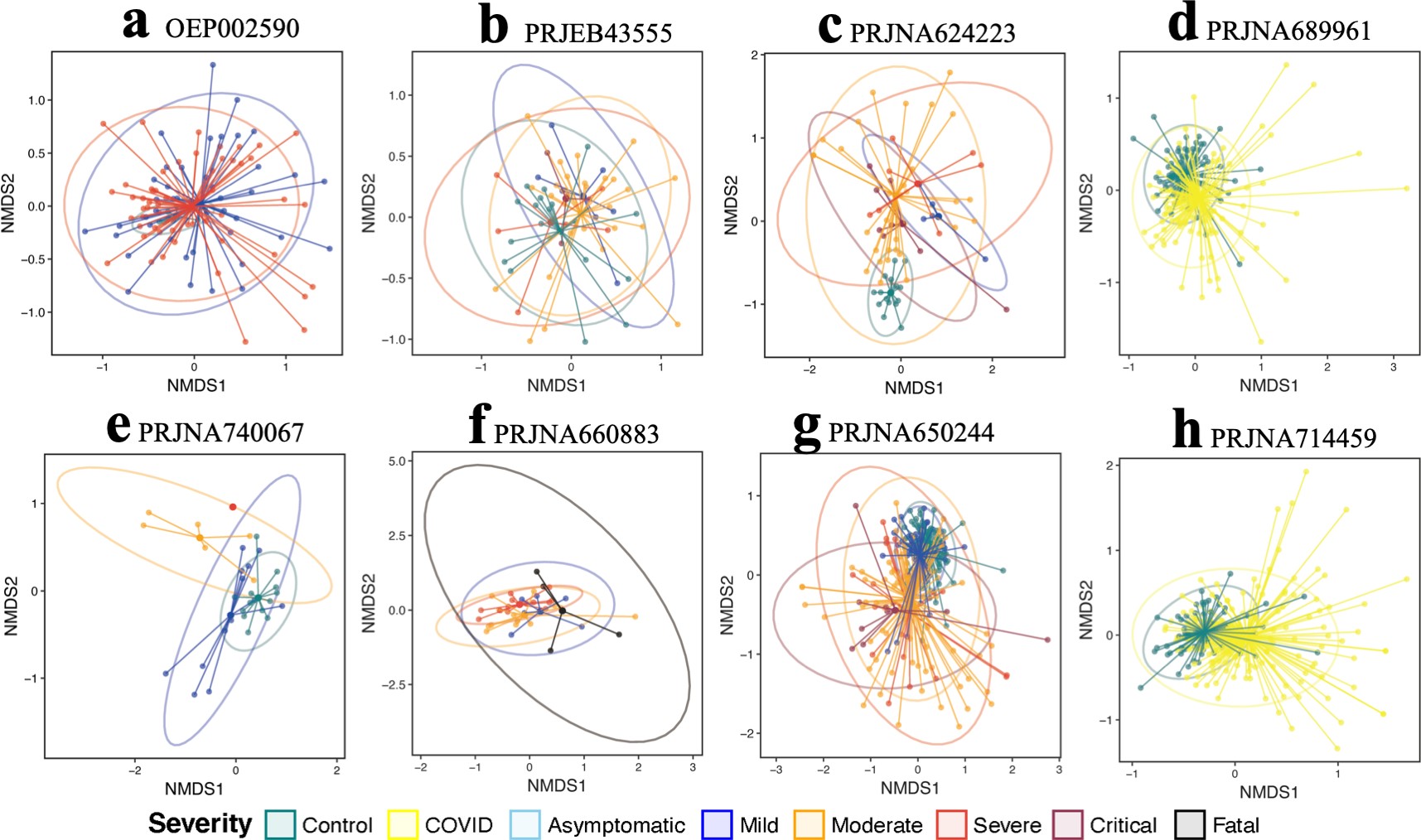
61

62

# Extended Data Fig. 2. Non-metric multidimensional scaling (NMDS) based on species-level Bray-

1. **Curtis dissimilarity of eight individual shotgun metagenome cohorts.** Colour key for severity
2. category is shown below the plots. COVID indicates uncategorized COVID severity. 66


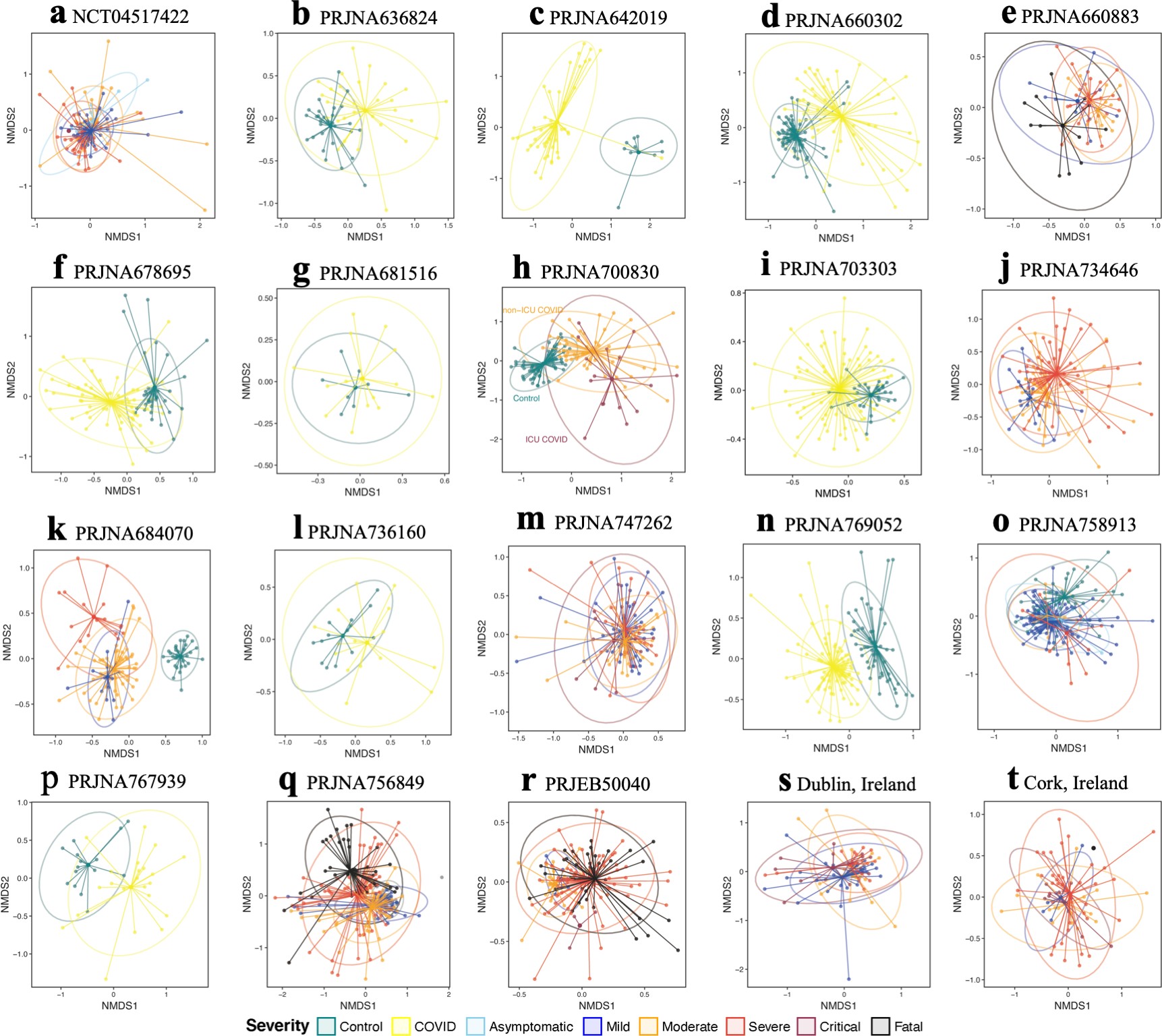
67

68

# Extended Data Fig. 3. Non-metric multidimensional scaling (NMDS) based on species-level Bray-

1. **Curtis dissimilarity of 20 individual 16S amplicon cohorts with different COVID disease severity**
2. **within cohort.** Colour key for severity category is shown below the plots. COVID indicates
3. uncategorized COVID severity. Cork, Ireland indicates PRJEB55909; Dublin, Ireland indicates 73 PRJEB55910.

74


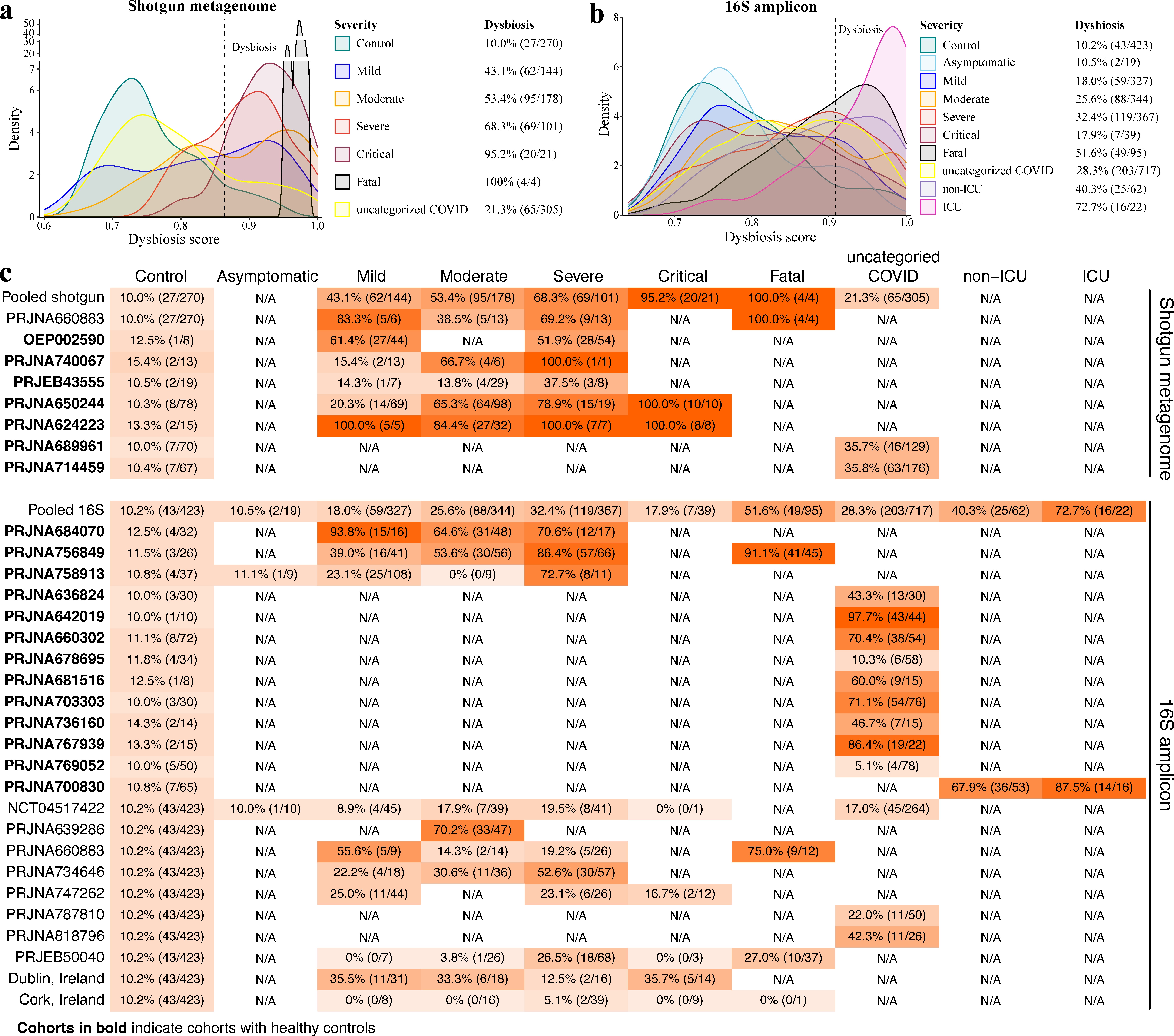
75

76

# Extended Data Fig. 4. Global distribution of microbial dysbiosis scores as a measure of COVID

1. **disease activity.** Density of microbial dysbiosis scores of (a) pooled Shotgun metagenomes (n=1,023)
2. and (b) pooled 16S rRNA gene amplicon sequencing samples (n=2,415); (c) Microbiome dysbiosis
3. frequency is associated with disease severity. For cohorts without healthy controls, microbiome
4. dysbiosis was estimated by comparing with data from the pooled healthy controls. Cork, Ireland
5. indicates PRJEB55909; Dublin, Ireland indicates PRJEB55910. 83


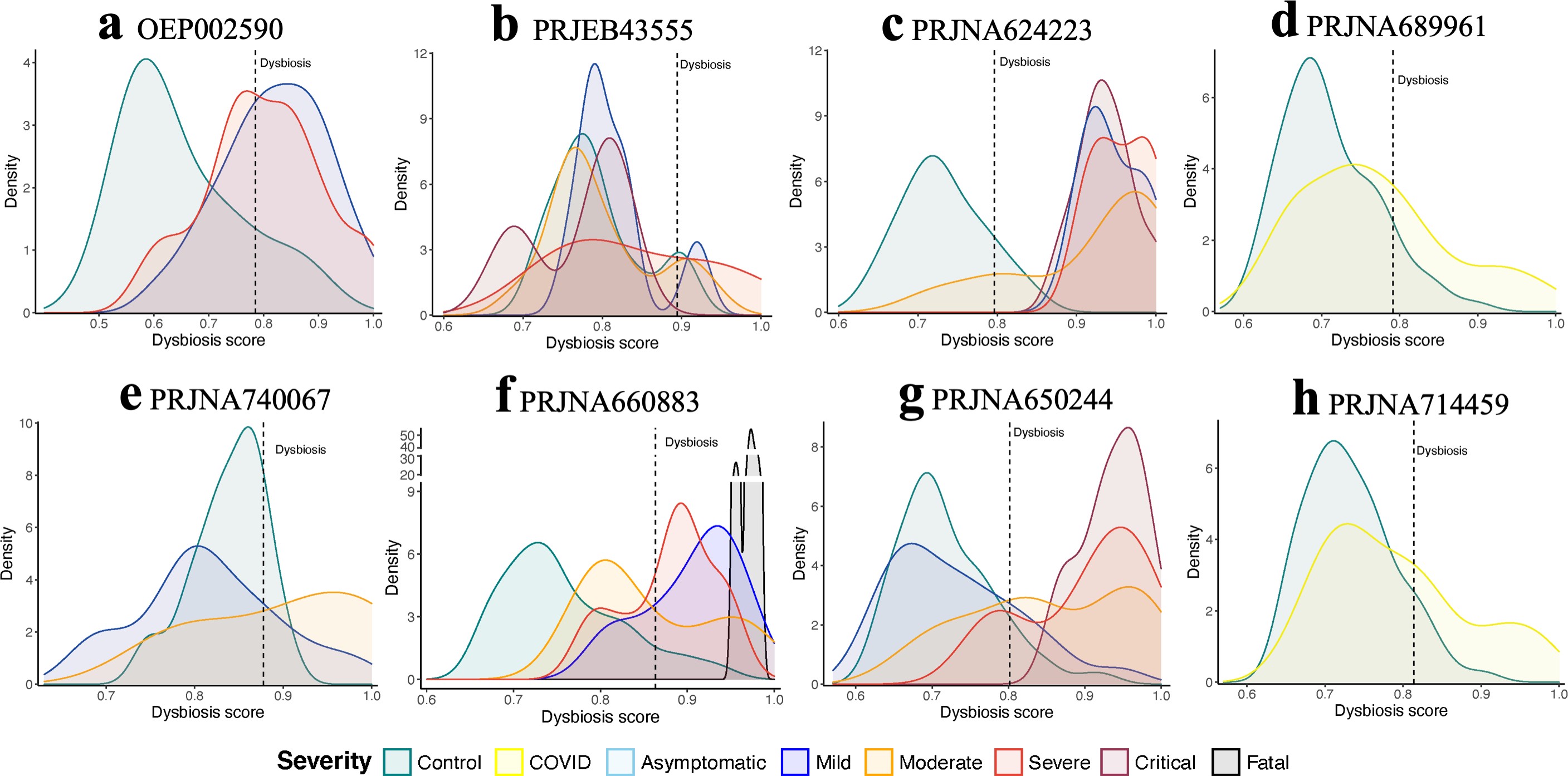
84

85

# Extended Data Fig. 5. Distribution of microbial dysbiosis scores as a measure of disease activity

1. **in individual shotgun metagenomic cohorts.** The dysbiosis score of (f) PRJNA660883 cohort was
2. compared with 270 global healthy controls (i.e., healthy controls from all other cohorts) as there was no
3. healthy control in this cohort. Colour key for severity category is shown below the plots. COVID
4. indicates uncategorized COVID severity. 91

92

93

94

95

96

97

98

99

100

101

102


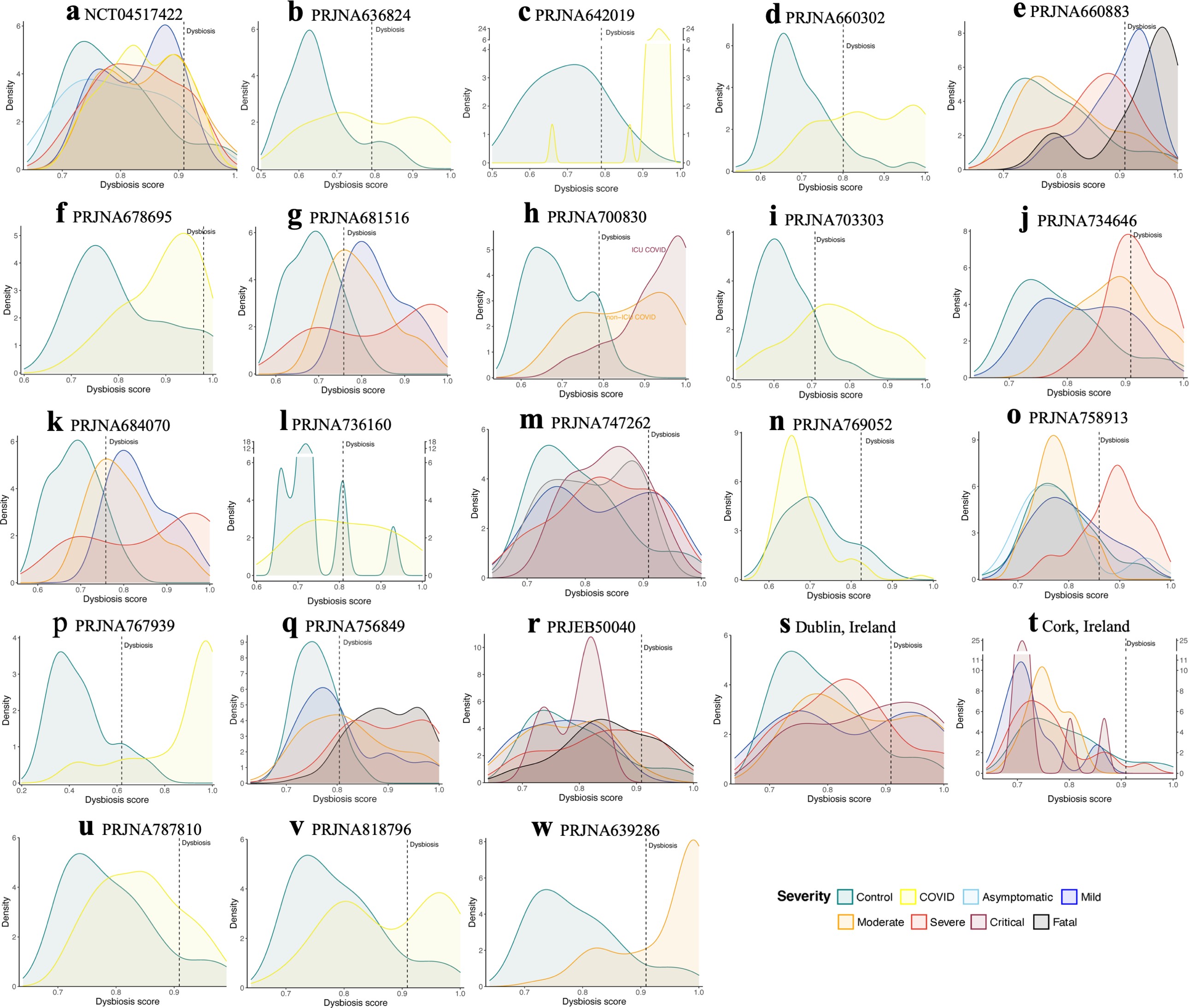
**Extended Data Fig. 6**. **Distribution of microbial dysbiosis scores as a measure of disease activity in individual 16S amplicon cohorts.** Dysbiosis scores of (a) NCT04517422, (e) PRJNA660883, (j) PRJNA734646, (m) PRJNA747262, (r) PRJEB50040, (s) Dublin Ireland, (t) Cork Ireland, (u) PRJNA787810, (v) PRJNA818796, and (w) PRJNA639286 cohorts were compared with 413 global healthy controls (i.e., healthy controls from all other cohorts) as there was no healthy control in these cohorts. Colour key for severity category is shown below the plots. COVID indicates uncategorized COVID severity. Cork, Ireland indicates PRJEB55909; Dublin, Ireland indicates PRJEB55910.

103

104

105

106

107

108

#
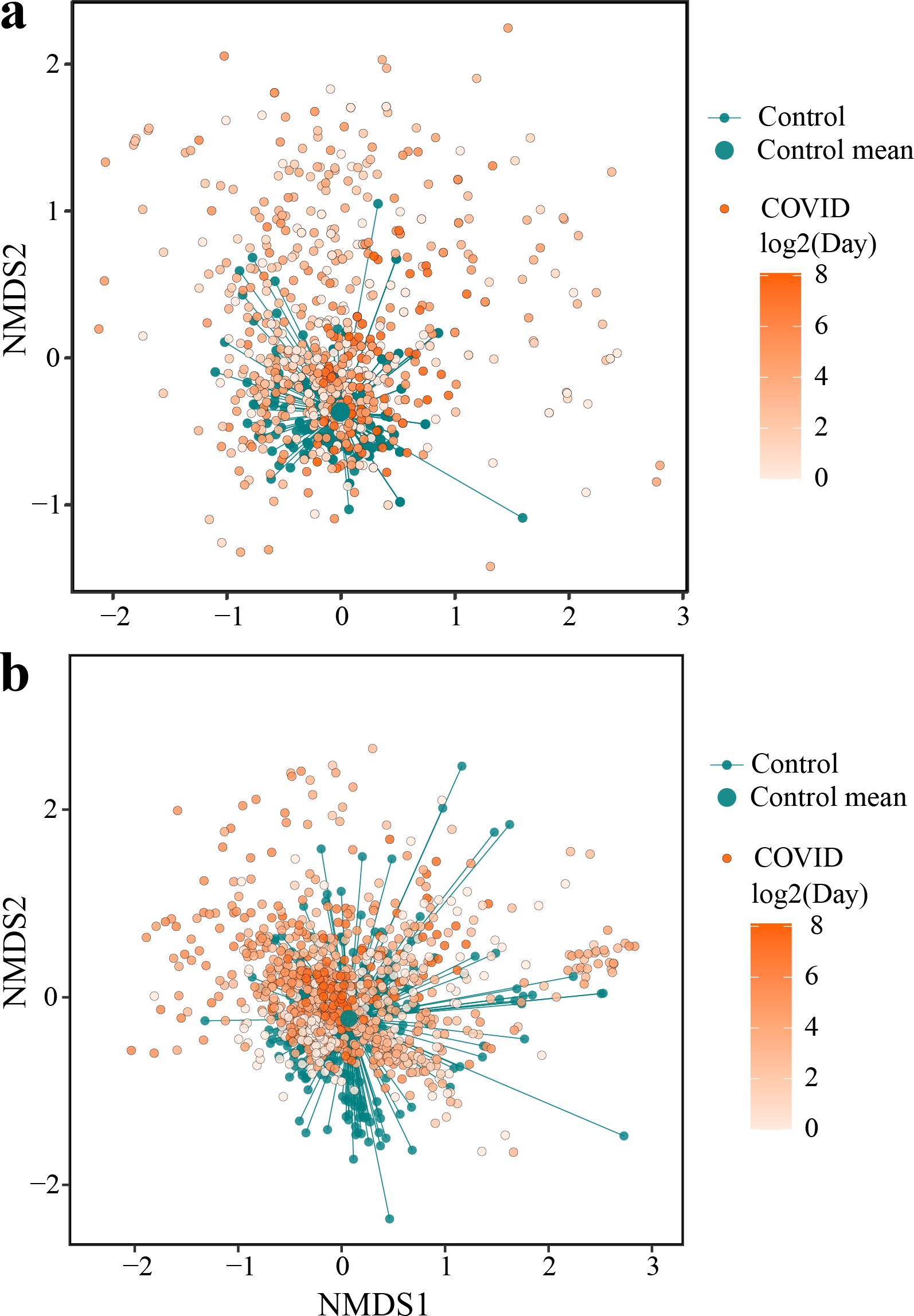
Extended Data Fig. 7. Non-metric multidimensional scaling (NMDS) based on species-level Bray- Curtis dissimilarity matrices from (a) pooled shotgun metagenomes (n = 753) and (b) pooled 16S rRNA gene amplicon sequencing samples (n = 1,302) with known sampling day.

109

110

111

112

113

114

115

116

117

118


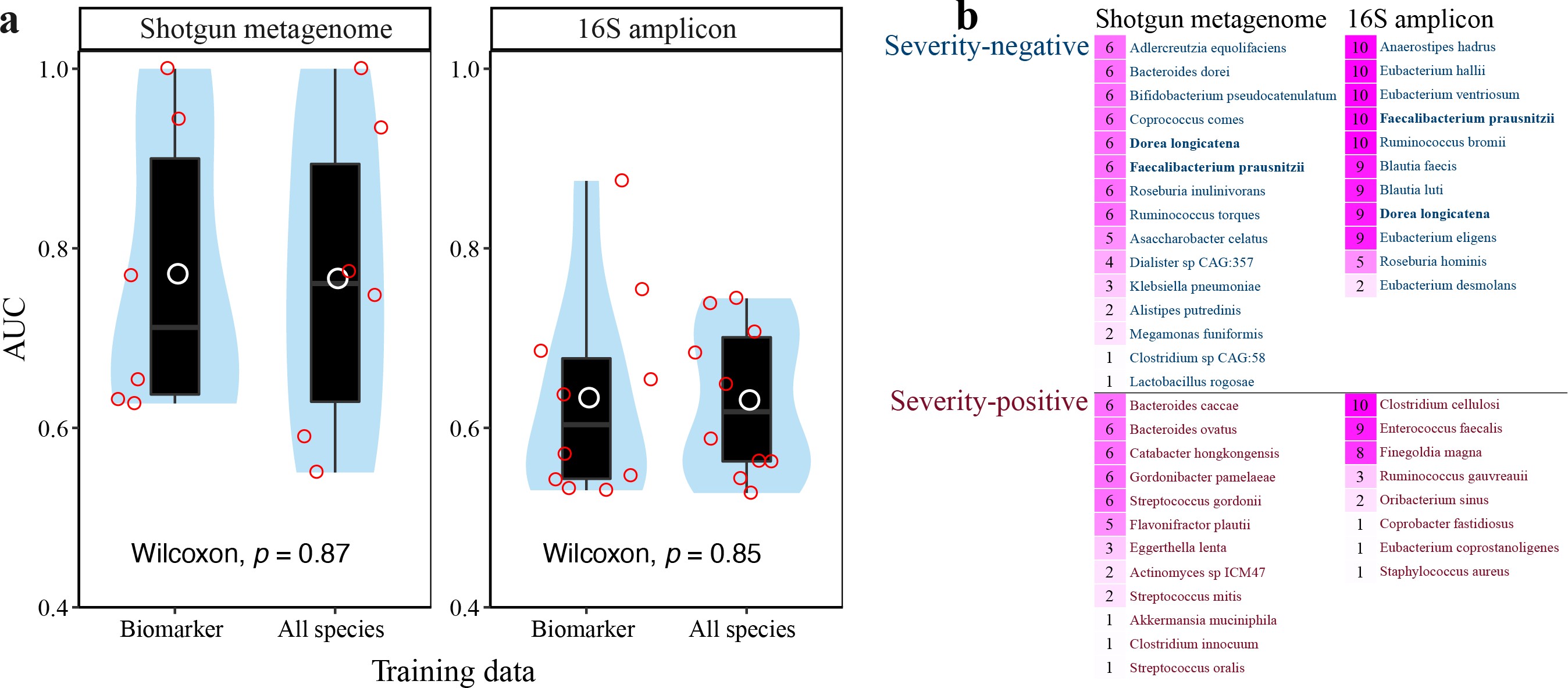
**Extended Data Fig. 8. Predictive accuracy of a random forest (RF) regression model based on biomarkers was comparable to that based on all species, irrespective of shotgun metagenomic or 16S amplicon data**. a. Predictive accuracy resulting from leave-one-out cross validation as measured by the area under the curves (AUCs). b. Number of cohorts where the top important features (74 for metagenomic data and 66 for 16S data) of the RF regression model overlapped with biomarkers identified in Fig. 3.

119

120

121

122

123

124

125

126

127


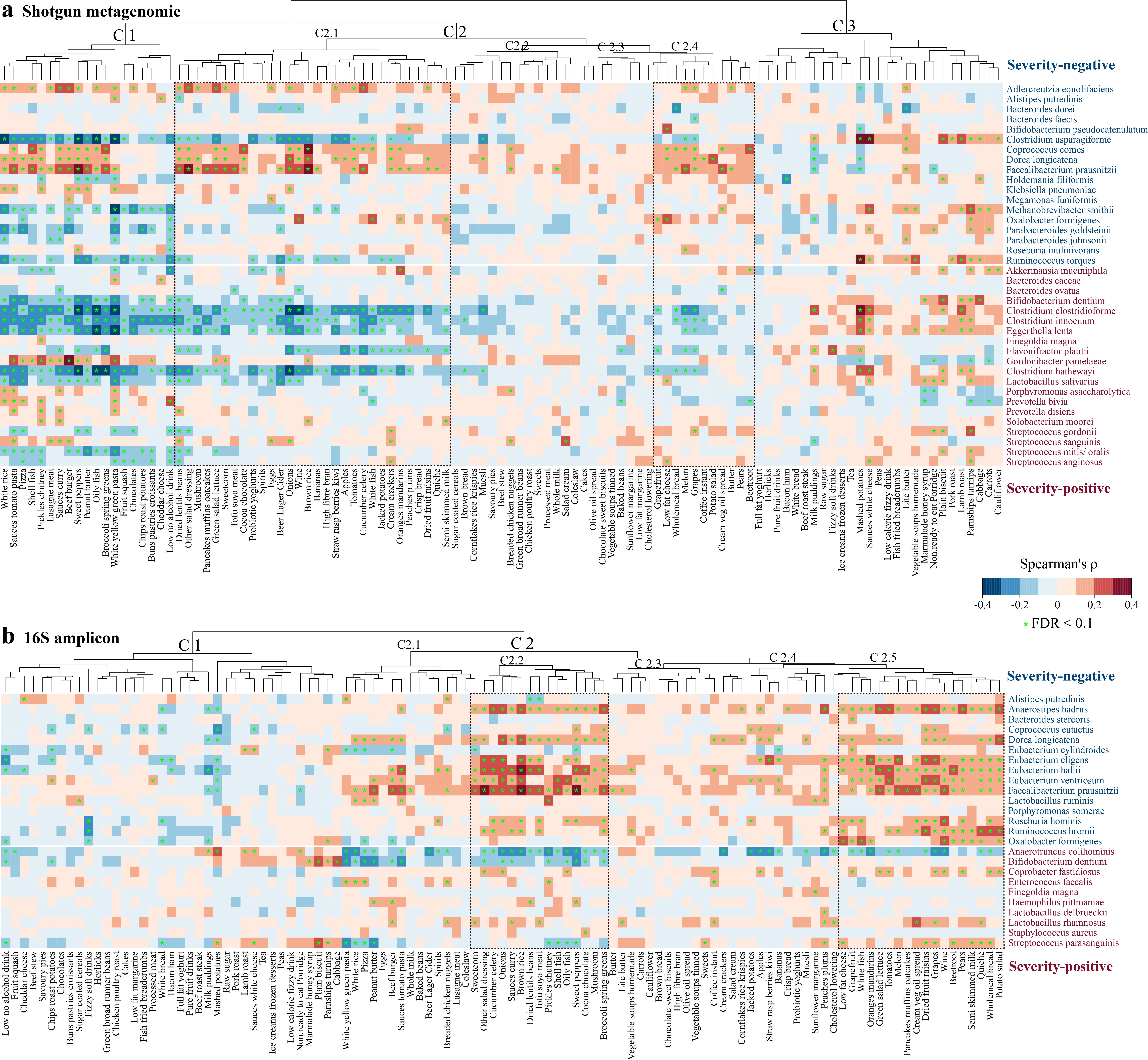
**Extended Data Fig. 9. Associations between dietary intake (assessed through food frequency questionnaires and parsed by the European food classification system) and gut microbiome biomarkers.** Detected (a) shotgun metagenomic and (b) 16S amplicon biomarkers in the healthy individuals of published datasets (see Methods). Cluster (C) was generated with heatmap.2 function using a complete agglomeration method based on Euclidean distance. The color gradient indicates Spearman's correlation coefficient (ρ) for each cohort; * indicates FDR adjusted *p* < 0.1.

80

70

60

Fungal read proportion (%)

50

40

30

20

10

0

Fungi (dummy088 PRJNA650244)

Candida glabrata (dummy088 PRJNA650244)

Fungi (CoV4 PRJNA624223) Candida glabrata (CoV4

PRJNA624223)

128

129

130

131

132

1 5 23

# Extended Data Fig. 10. Fungal read proportion in the gut metagenome of two subjects over time.

Color indicates days since diagnosis.
